# Randomised controlled trial and economic evaluation of a task-based weight management group programme

**DOI:** 10.1186/s12889-019-6679-3

**Published:** 2019-04-02

**Authors:** Hayden McRobbie, Peter Hajek, Sarrah Peerbux, Brennan C. Kahan, Sandra Eldridge, Dominic Trépel, Steve Parrott, Chris Griffiths, Sarah Snuggs, Katie Myers Smith

**Affiliations:** 1 Health and Lifestyle Research Unit, Wolfson Institute of Preventive Medicine, Queen Mary University of London, Health and Lifestyle Research Unit, 2 Stayner’s Road, London, E1 4AH UK; 2 Pragmatic Clinical Trials Unit, Queen Mary University of London, Barts and The London School of Medicine and Dentistry, 4 Newark Street, London, E1 2AT UK; 3 Department of Health Sciences, The University of York, Seebohm Rowntree Building, Heslington, York, YO10 5DD UK; 4 Centre for Primary Care and Public Health, Blizard Institute, Queen Mary University of London, Barts and The London School of Medicine and Dentistry, 4 Newark Street, London, E1 2AT UK

**Keywords:** Weight management, Obesity, Weight loss, Cost-effectiveness

## Abstract

**Background:**

Obesity is a rising global threat to health and a major contributor to health inequalities. Weight management programmes that are effective, economical and reach underprivileged groups are needed. We examined whether a multi-modal group intervention structured to cater for clients from disadvantaged communities (Weight Action Programme; WAP) has better one-year outcomes than a primary care standard weight management intervention delivered by practice nurses (PNI).

**Methods:**

In this randomised controlled trial, 330 obese adults were recruited from general practices in London and allocated (2:1) to WAP (*N* = 221) delivered over eight weekly group sessions or PNI (*N* = 109) who received four sessions over eight weeks. Both interventions covered diet, physical activity and self-monitoring. The primary outcome was the change in weight from baseline at 12 months. To indicate value to the NHS, a cost effectiveness analysis estimated group differences in cost and Quality-Adjusted Life-Years (QALYs) related to WAP.

**Results:**

Participants were recruited from September 2012 to January 2014 with follow-up completed in February 2015. Most participants were not in paid employment and 60% were from ethnic minorities. 88% of participants in each study arm provided at least one recorded outcome and were included in the primary analysis. Compared with the PNI, WAP was associated with greater weight loss overall (− 4·2 kg vs. − 2·3 kg; difference = − 1·9 kg, 95% CI: -3·7 to − 0·1; *P* = 0·04) and was more likely to generate a weight loss of at least 5% at 12 months (41% vs. 27%, OR = 14·61 95% CI: 2·32 to 91·96, *P* = 0·004). With an incremental cost-effectiveness ratio (ICER) of £7742/QALY, WAP would be considered highly cost effective compared to PNI.

**Conclusions:**

The task-based programme evaluated in this study can provide a template for an effective and economical approach to weight management that can reach clients from disadvantaged communities.

**Trial registration:**

ISRCTN ISRCTN45820471. Registered 12/10/2012 (retrospectively registered).

**Electronic supplementary material:**

The online version of this article (10.1186/s12889-019-6679-3) contains supplementary material, which is available to authorized users.

## Background

Obesity is a rising global threat to health [1] with concomitant increases in social, medical, and public health costs. In the UK 10% of morbidity and mortality in the UK [2] can be attributed to obesity and contributes to health inequalities, with rates higher in lower socioeconomic groups [3]. The picture in other developed countries is similar [4].

When compared to usual care, brief interventions in primary care that encourage people to enrol in a weight management service are found to provide approximately 1.5 kg weight loss within a year, [5]. In people unable to lose weight on their own, surgical interventions can be highly effective, but they are associated with considerable costs and cannot be easily provided to large numbers of people [6]. Existing pharmacological treatments have more limited effects [7]. Stand-alone dietary interventions have only limited effects [8], and brief routine interventions within primary care show very little change [9]. Behavioural interventions that are more intensive show minimal but sustainable weight loss [10]. The most recent meta-analyses of behavioural approaches reported a pooled mean difference in weight loss for intervention versus control comparisons at 12 months of 2·8 kg (95% CI 2.1 to 3·6 kg) [11], while in trials conducted in an ‘everyday context’ via commercial weight loss programmes the difference in weight loss was 2·2 kg (95% CI 1·5 to 2·9 kg) [12]. These effects are modest but sustainable, and they are sufficient to lead to long-term health improvements [13].

The majority of studies in this field have focused on participant groups from middle to high socioeconomic groups and disadvantaged and ethnic minority groups, that have increased risk for obesity, are rarely represented [14]. This is the case particularly for multi-component lifestyle treatments that typically place considerable demands on participants’ engagement, understanding and commitment and sometimes also on their resources. There are however exceptions. For example, around 73% of participants in the Lighten Up study were from the bottom two quintiles of deprivation [15]. Another is the Counterweight programme, which has been implemented in a range of healthcare settings in the UK and enrolled people from a mix of social and ethnic backgrounds. Evaluations of this programme [16–18] have reported weight loss of ≥5% in 30–35% of people that completed 12-month follow-up. However, this programme has not been evaluated in a randomized controlled trial.

There is a need for weight management programmes that are effective and attractive for patients from disadvantaged communities. Basing a weight management course on simple and manageable tasks that are regularly monitored can address some of the problems that complex multicomponent programmes can pose. The present trial aimed to determine the clinical- and cost-effectiveness of targeting a disadvantaged population with a multi-modal task-based group intervention (Weight Action Programme), which has shown encouraging short-term results previously [19], has an effect at one year that is better than a ‘best practice’ weight management intervention provided by practice nurses.

## Methods

### Study design

We conducted a two-arm, parallel-group randomised controlled trial, with 12 months follow-up, in general practices in the London inner city boroughs of Tower Hamlets and Hackney, the two most economically deprived boroughs in London [20]. Ethical approval was granted from the London - Central Ethics Committee (ref: 12/LO/0122). The study protocol can be accessed via https://www.journalslibrary.nihr.ac.uk/programmes/hta/0912734#/ and the funder report contains further details on the study [21].

### Participants

We recruited adults (aged ≥18 years) living in the study areas who had BMI ≥ 30 kg/m2 or a BMI of ≥28 kg/m2 with co-morbidities (criteria for referring patients for weight loss interventions in the UK). Exclusion criteria included not speaking English, BMI > 45, losing > 5% of their body weight in the previous 6 months, pregnancy, and currently taking a psychiatric medication (because of medication effects on weight). No other co-morbidities were excluded to ensure that the study addressed the needs of the National Health Service (NHS) and the results are generalisable to target populations.

Participants were recruited via fax referrals, posters, flyers, and mailshots at two large local GP practices (one in each borough), via referral from four neighbouring GP practices, posters and leaflets at local community venues, and three advertisements in the local press.

### Randomisation and masking

Participants were randomised 2:1 (WAP:PNI) using permuted blocks which randomly varied in size (18, 21, and 24) and were stratified by the two GP sites. Randomisation was conducted using an independent online server based at the University of Sheffield. Investigators randomising participants were blind to the allocation until they performed the randomisation. Staff collecting outcomes were blind to participant allocation.

### Procedures

At first contact (via telephone) participants received information about the study and were screened for eligibility. Eligible participants were sent (via post or email) the participant information sheet, the baseline questionnaire, and an invitation to the first study session a few days later. At this session participants provided written informed consent and baseline assessment was undertaken. A few days later participants attended to complete questionnaires and provide other measures and to be randomised. The first session of WAP and PNI took place 7–14 days after randomisation.

Both interventions were delivered over eight weeks, with follow-ups scheduled at 6 and 12 months post-randomisation. Participants were recruited from September 2012 to January 2014 with 1-year follow-ups completed in February 2015. Participants invited to attend follow-up were offered £10 toward travel expenses.

### Interventions

#### Weight action Programme (WAP)

WAP is a task-based multi-modal group intervention that over a number of years has been modified through client feedback to tailor it to appeal to disadvantaged groups and has produced encouraging results in two pilot studies [19].

WAP aims to provide clear and simple advice on diet, physical activity and self-monitoring via a range of concrete and verifiable tasks agreed individually with each participant, as opposed to providing written and verbal advice as is typically done (see Practice Nurse Intervention). Where printed information is provided, it is mostly in pictorial and a simple English format making it more accessible to clients whose first language is not English or who have lower levels of education. The main innovative feature of the programme is that participants aim to complete a number of tasks that are monitored via ‘task cards’ marked every day for at least one week. Participants can choose not to continue with the task if they found it unhelpful, but they commit to trying it first for one full week. The tasks include increasing pedometer targets gradually up to 10,000 steps per day (participants were provided with an Oregon pedometer PE980), using a food diary, removing triggers to eating from the environment, implementing ‘easy switches’ (food swaps), monitoring hunger levels before and during eating, exercising three times a week, recording instances of saying “No” to unnecessary food and monitoring weight. Participants also receive information about using orlistat (see below). Sessions also include imparting and testing the knowledge of caloric content of food. The other key feature of WAP is the use of a group format focusing on social support.

The programme comprises 8 weekly group sessions lasting one hour each, followed by optional monthly group meetings. Two advisors trained in WAP delivery conducted the sessions in groups of 10 to 21 participants.

#### Practice nurse intervention (PNI)

We sought to compare WAP with a ‘best practice’ intervention that could be undertaken in a Primary Healthcare setting and, in seeking to identify current best-practice, we conducted a survey of weight management interventions in six GP surgeries in 2011. Most GPs provided brief advice and they were then referred on to speak with a practice nurse. Most nurses provided one -off sessions with some follow-up, which was typically over a two to eight week period. A referral to community-based physical activity programmes was offered in half the surveyed practices.

The PNI incorporated all of the suggested practices to mimic a more intense model of “best practice”. The nurses were trained to give the intervention which took place over eight weeks (4 sessions in total), with the intervention incorporating national [22] and NHS guidelines.

The nurse provided advice on (1) Diet, e.g. basic introduction to different food groups, how to read food labels and identify calories in food; limiting the size of the portions of food eaten; and choosing healthier options; (2) Activity, e.g. finding exercise options they will enjoy and can do each day; minimise sitting, watching TV (sedentary activities); encouraged to go to local exercise classes/activities; and (3) Self-monitoring, i.e. keeping track of eating habits with a food diary, using a pedometer (this was not provided), and taking weight at home. Participants also received a recommendation to use orlistat (see below).

The advice was accompanied by the standard set of NHS ‘Change4Life’ leaflets [23].

Each session lasted up to 30 min.

### Both interventions

Both study arms received written information about food labels and a guide to local physical activity opportunities. All subjects were given an adapted version of information on orlistat on the NHS website [24], which at the time of the study was the only pharmaceutical option for weight loss. It combines physiological effects and effects on behaviour change (avoiding dietary fat). If they wished to use orlistat they had to make contact with their GP.

We measured weight in participants at every face-to-face visit.

Participants were asked to report any other method used to manage their weight throughout the study period.

Details of the monitoring of the fidelity of each intervention is provided in Additional file 1.

### Measures

Measurements were taken at baseline, randomisation, throughout and at the end of 8 weeks of treatment, and at the 6 and 12-month follow-up. The following variables were collected at baseline: Demographic details, health status, weight loss history, concurrent medications, height and weight. Waist circumference and blood pressure (using Omron 705IT BP monitor) were collected at the randomisation session. The International Physical Activity Questionnaire (IPAQ) [25] was administered at baseline and the Food craving inventory (FCI), [26] Use of health services questionnaire, Three Factor Eating Questionnaire (TFEQ) [27], EQ-5D-5 L (a measure of health outcomes) [28], and a picture-based Food Knowledge Assessment (FKA), a tool developed at the Health and Lifestyle unit to assess knowledge of caloric content of different food groups were administered at the randomisation session.

At 2, 6 and 12 months, weight, waist circumference, blood pressure, participant feedback, use of any concomitant weight loss treatment, IPAQ, FKA, FCI (also measured at 1 month), ratings of the intervention received and adverse events were collected. In addition, the TFEQ, EQ-5D and use of health services were collected at the 6 and 12-month follow-up visits.

### Outcomes

The primary outcome was the change in weight from baseline at 12 months. Secondary outcomes included proportion of participants losing 5% of body weight, changes in weight at 2 and 6 months, changes in BMI, changes in waist circumference and blood pressure, and changes in food knowledge and questionnaire scores.

### Statistical analysis

Based on data from similar populations and interventions, we predicted weight loss of WAP 3 kg vs. PNI 0·4 kg in participants who were contacted, with no difference between treatment arms for participants who were not available for contact. With 50% of participants available for follow-up (a conservative estimate), the weight loss difference would be 1·3 kg (WAP 1·5 kg vs. PNI 0·2 kg). With SD = 3 in both treatment arms and *p* < 0.05 (two-sided), 112 participants in each arm would be needed to detect this difference with 90% power. To minimise clustering effects due to the group intervention (mean cluster size of 18 and an intra-cluster r = 0·05), 208 participants would be needed in the WAP arm and 108 in the PNI arm. We increased this to 220 and 110 participants to achieve an allocation ratio of 2:1. We therefore aimed to recruit 330 participants.

All analyses were performed using intention-to-treat (ITT) principle. Participants recorded with at least one outcome were included in the study arm to which they were randomised [29]. Analyses accounted for clustering in both intervention (group or nurse, depending on treatment arm) [30]. Cluster effect was entered as a random intercept as part of a mixed-effects regression model. All analyses were adjusted for baseline weight, age, gender, ethnicity, smoking status, and GP practice as covariates [31, 32]. Missing baseline data were entered for analysis using mean imputation [33].

A mixed-effects linear regression model was used to analyse the primary outcome. Weight change at 1, 2, 6 and 12 months was included in the model and included a random intercept for ‘cluster’ (group or nurse, depending on treatment arm). An unstructured correlation structure, using restricted maximum likelihood, was used to model the correlation between data at different time points from the same participant. Fixed factors included treatment arm, time point (included as an indicator variable) and their interaction. Covariates mentioned above were also included in the model as fixed factors. A Kenward-Roger degree-of-freedom correction was used.

Several sensitivity analyses were undertaken to check the robustness of our assumptions regarding missing data and to check if there was any impact on results of participants who were identified as pregnant or had bariatric surgery during the follow-up period (details of these sensitivity analyses and the analysis of all secondary outcomes are available in the online Additional files 2 and 3).

An online Oracle database was used to enter the data. Stata (version 14) was used for all analyses.

We estimated NHS health care costs from sources that included NHS reference costs [34] and Personal Social Services Research Unit’s (PSSRU) Costs of Health and Social Care [35]. Participants’ responses to the EQ-5D-5 L questionnaire were used to estimate health states utilities using the UK value set [36] and Quality-Adjusted Life Years (QALYs) were estimated across all timepoints. No discounting was needed as participants were followed-up post-randomisation for 12 months. As per NICE guidance, the primary endpoint of the cost-effectiveness analysis were costs, QALYs and the Incremental Cost Effectiveness Ratio (ICER) (i.e. the ratio of the incremental costs of the intervention (versus control) over the incremental effective (versus control). To determine the level of uncertainty in the joint distribution of cost and QALYs from the two arms of the study, results were bootstrapped using 10,000 replications and results were presented on a cost-effectiveness acceptability curves.

## Results

**Fig. 1:**
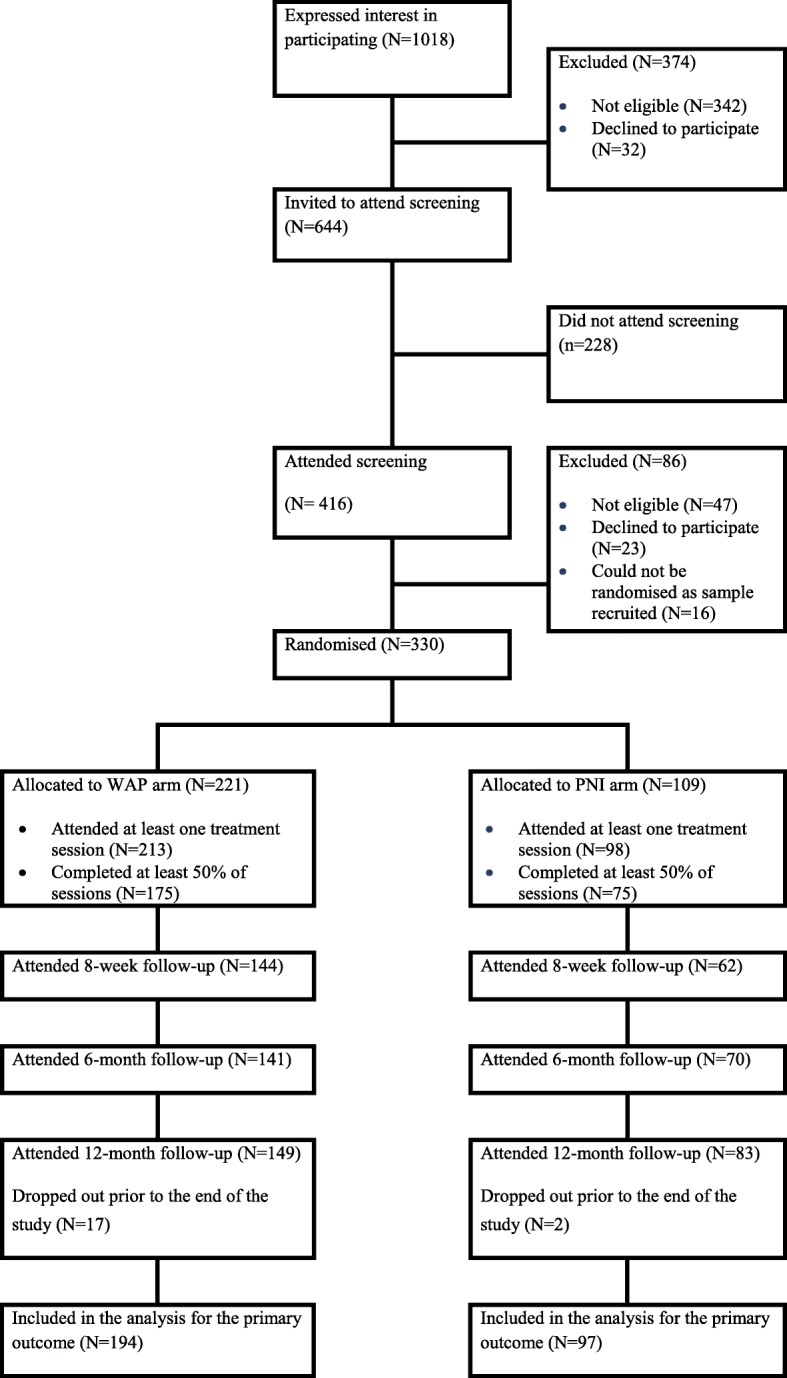
CONSORT diagram

Figure 1 shows participant flow through the trial. Participants were recruited from 24/09/2012 to 31/01/2014. 221 participants were allocated to the intervention group and 109 to the control group. A total of 291 participants provided at least one recorded primary outcome measurement (PNI *N* = 97 (89%); WAP *N* = 194 (88%)).

Table 1 shows participant characteristics. Reflecting the intended target population, most participants were not in paid employment and some 60% belonged to ethnic minorities.

**Table 1:** Sample characteristicss

	PNI (*n* = 98–109)^a^	WAP (*n* = 197–221)^a^
Age (years): mean (SD)	45·1 (14·2)	46·6 (15·0)
Female: *N* (%)	75 (68.8)	161 (72.9)
Married or living with partner *N* (%)	49 (44.9)	92 (41.6)
Ethnicity: White British *N* (%)	46 (42.6)	85 (38.0)
Education: A-Level and above *N* (%)	53 (49.1)	101 (45.7)
In paid employment *N* (%)	57 (52.3)	103 (46.6)
Entitled to free prescriptions: *N* (%)	62 (56.9)	133 (60.2)
Current smoker: *N* (%)	18 (16.5)	35 (15.8)
Weight (kg): mean (SD)	98·3 (16·6)	95·5 (15·8)
BMI: mean (SD)	35·7 (4·3)	35·0 (4·2)
Waist circumference: mean (SD)	114·2 (10·1)	113·4 (10·7)
Systolic blood pressure: mean (SD)	134·8 (15·9)	134·5 (16·7)
Diastolic blood pressure: mean (SD)	80·6 (8·6)	81·3 (10·5)
Number of previous attempts at weight loss: median (IQR)	3 (2, 5)	3 (1, 5)
Greatest previous amount of weight loss: median (IQR)	9·3 (5·0, 19·1)	10·9 (6·0, 19·1)

^a^*N* varies due to missing data

A median of 24 participants were allocated per practice nurse (4 nurses in total). A median of 15 participants attended the group (15 groups in total). A high number in both interventions attended at least half of the treatment sessions (79% WAP versus 69% PNI participants), with 65 and 57% of participants attending the final session at 8-weeks. Very few attended the WAP maintenance sessions (one in five).

Attendance for follow up was high, with 70% of participants followed-up at 12 months. Participants in the PNI group had slightly increased follow-up rates than the WAP arm (76% vs. 67% respectively).

The primary outcome analysis showed that participants in the WAP arm lost significantly more weight (4·2 kg, SD = 7·3) than those in the PNI arm (2·3 kg, SD = 6·6): 95% CI: -3·7 to − 0·1; *p* = 0·04. At all time points (except 1-month post randomisation), there was a significantly greater amount of weight lost in the WAP compared with the PNI arm (see Table 2).

**Table 2:** Mean weight loss over 12 months

	PN PNI arm Mean (SD)^a^	WAP arm Mean (SD)^a^	Treatment effect (95% CI)^b^	*P*-value
2 months	−2·2 (2·6)	-3·2 (2·7)	-1·0 (−1·7, −0.3)	0·009
6 months	-2·1 (4·3)	−5·0 (5·4)	−2·5 (−3·8, −1.2)	< 0·001
12 months	− 2·3 (6·6)	−4·2 (7·3)	−1·9 (− 3·7, − 0.1)	0·04

^a^The total sample used was based on those with a documented weight measurement at each follow-up time-point. For the PNI arm this was 62, 70, and 83 at 2, 6 and 12 months respectively. For the WAP arm it was 144, 141, and 149

^b^For the treatment effect, which was calculated using a mixed-effects regression model, the total sample was 97 and 194, for the PNI and WAP arms, respectively

**Fig. 2:**
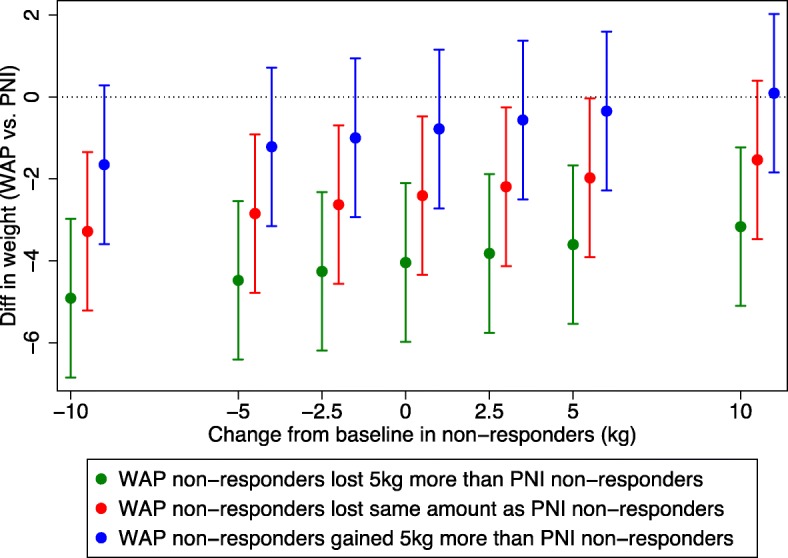
Treatment effect and 95% CI for change in weight at 12 months under the assumption that non-responders are missing-not-at-random

The results did not change when we used a model that assumed that data were not missing at random. An exception to this was if it was assumed that WAP participants who were lost to follow-up gained more weight than those lost to follow-up in the PNI arm, or all lost to follow-up in either arm had significant weight gain (i.e. 10 kg). When we used baseline observation forward in all those lost to follow-up, a similar result was seen (i.e. a greater weight loss in the WAP arm vs. PNI − 2·4 kg; 95% CI -4·3, − 0·5) (See Fig. 2).

Table 3 shows the results of the complete case analysis. Participants were included who provided data at 12 months, and an analysis was done which excluded those pregnant or who had undergone bariatric surgery, after randomisation which did not substantially alter the treatment effect.

**Table 3:** Sensitivity analyses for primary outcome

	PNI arm Mean (SD)	WAP arm Mean (SD)	Treatment effect (95% CI)
Complete case analysis (*N* = 232)	−2.3 (6.6)	−4.2 (7.3)	− 2.4 (− 4.9, 0.1)
Excluding participants who had bariatric surgery or became pregnant during follow-up^a^ (*N* = 221)	− 2.1 (5.7)	−4.2 (7.3)	−2.1 (− 3.9, − 0.4)

^a^11 excluded: gastric bypass (PNI 1 (lost 34.2 kg), intervention 0), pregnancy (PNI 5, intervention 5)

Among those participants with a recorded outcome at 12-months, a greater proportion in the WAP arm had lost at least 5% of body weight, compared with participants in the PNI arm (41% [61/149] vs. 27% [22/83], *p* = 0·004). Waist circumference changes favoured the WAP arm, but not significantly so (− 4·1 cm vs-2·0, *p* = 0·07). There were no differences between the study arms in changes in blood pressure. Participants in the WAP arm showed a significant increase in their knowledge of the calorie content of foods at the end of treatment and at 6-months, but by 12-months this effect had disappeared. WAP participants used orlistat more than participants in the PNI arm (31% vs. 6%; OR = 6·50; 95% CI: 2·78 to 15·59; *p* < 0·001) and for those who used orlistat their weight loss was increased at 12 month follow up (mean − 5·4 kg, SD = 8·1) compared with those who did not (mean − 2·9 kg, SD = 6·6; 95% CI for the difference:-4·5 to − 0.4; *p* = 0·02).

Physical activity levels in both groups increased, with the WAP arm showing a marginally greater increase (359 vs. 215 MET-minutes/week, 95% CI: -312 to 153; *p* = 0·18).

At the end of treatment, ratings of helpfulness of both interventions were high, but WAP received a higher endorsement (9·1 vs. 8·0, *p* < 0·001) and WAP participants were also more likely to recommend the programme to others (9·3 vs. 8·1, *p* < 0·001). The differences were maintained at 12 months (8·4 vs. 7·2, *p* = 0·001 and 8·8 vs. 7·8, *p* = 0·004, respectively).

The full set of secondary outcomes is provided in Additional file 2.

**Fig. 3:**
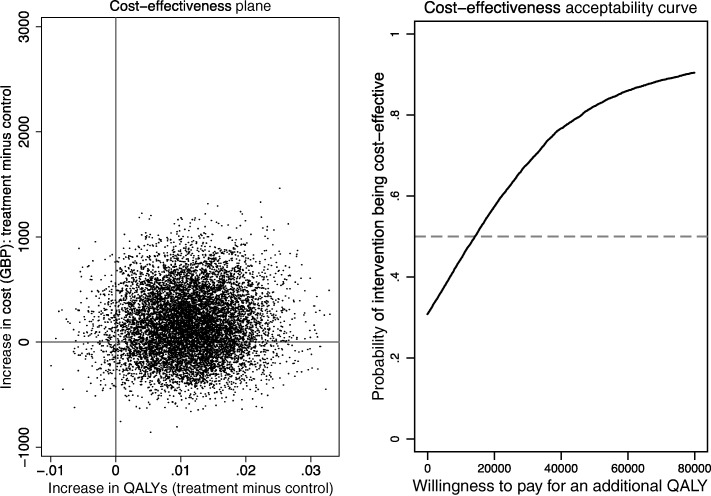
Cost effectiveness of WAP vs. PNI intervention

As the number of clients in each group were similar, the cost of WAP per client (£195) was similar to the cost of the PNI (£176). WAP resulted in a mean incremental gain in QALYs (controlling for baseline utility and age) of 0·0104 (95% CI: -0·0015 to 0·0224, *p* = 0·088) and non-significant mean incremental total cost was £80 (95% CI: -£505 to £667, *p* = 0·787). The base case incremental cost effectiveness ratio was £7742/QALY indicating that WAP, compared to PNI, is likely to represent value for money in the NHS. Based on the power available in this study, the probability of falling within the NICE threshold for reimbursement (£20,000 - £30,000) was between 68 and 77% (Fig. 3).

## Discussion

With no significant increase in cost, WAP generated greater weight loss than PNI. This difference between groups was evident by the end of the treatment period and maintained at one year. The results were not attributable to the control group performing poorly, as the PNI appears to have outperformed results in most previous studies [37]. In an evaluation of the Counterweight Programme [18], 10% of all eligible people lost ≥5% of baseline body weight at 12-month follow-up. In this trial, 27% of people in the PNI has achieved ≥5% weight loss (41% in the WAP arm). The differences were robust across different sensitivity analyses. As a results of being cost neutral but improving overall health-related quality of life, WAP appears to be highly cost-effective and this study provide a good level of certainty WAP would represent value for money, if deployed within the UK NHS.

The trial had several limitations. Although retention rates were comparatively good (e.g. Jebb et al. reported a retention rate of 61% at 12 months [38]), there were 30% of participants at one year who we were unable to contact. ‘Last observation carried forward’ is often used to address missing data but one limitation is it may overestimate treatment effects due to people usually engaging with a treatment while it is working but stop when not. We used mixed-effects model approach and several sensitivity analyses that confirmed the main result. Dropout rates were also similar in the two study arms. Nevertheless, no approach resolves the missing data problem completely.

As with other weight management programmes and trials, the participants were predominantly women and this may limit the generalisability of the results across genders. We also excluded those who could not speak English as the WAP programme includes group interaction, and this also limits the generalisability of the results. The simple and clear tasks however made the programme suitable for participants with English as a second language.

Trials of behavioural interventions cannot be blind and expectations may have affected the results if e.g. participants who believe that they were allocated to inferior treatment are more likely to drop out or participants in ‘active’ condition receive more attention and encouragement during post-treatment follow-ups. We tried to mitigate such effects by presenting the trial as studying two potentially useful interventions with no indications of expecting any of them to be superior; and ensuring that staff collecting outcomes at 6 and 12-month follow-up were blind to participant allocation. It is reassuring that attendance and follow-up rates in the two study arms were similar.

The sample included a high proportion of participants from ethnic minorities and participants with limited socioeconomic resources. Weight management studies, including those conducted in primary care [39], typically attract middle class participants and disadvantaged groups are normally under- represented [14]. The inclusiveness of this trial can be seen as a positive factor in evaluating an intervention suitable for use with under-served populations.

There were no significant change in blood pressure, this is in line with previous studies. A meta-analysis which included data from 34 trials, found no change in either systolic blood pressure with weight loss (18 trials) nor with diastolic blood pressure (21 trials) [40].

The weight loss achieved was modest, as is common for all existing weight management approaches with the exception of bariatric surgery (the one participant in the PNI arm who had the procedure lost 34 kg). However, the programme helped over 40% of participants to achieve a weight loss of at least 5% and this is expected to convey important health benefits [13]. Programme participation may also generate long-term lifestyle changes. PNI participants achieved their weight loss within the treatment period and maintained it, while WAP participants continued to lose weight even after the treatment period ended, although there was some weight regain over the last six months of follow-up.

There are several candidate explanations for WAP effects. The WAP condition offered more contact time, and there is a belief in the weight management field that more intensive interventions are more effective. However, this is not supported by data. A comprehensive meta-analysis found no evidence that more frequent contact generates greater weight loss [11]. This is similar to findings concerning intensity of contact in trials of behavioural counselling for smoking cessation [41]. One possible ‘active ingredient’ of WAP is the group format providing opportunities for observing other group members benefiting from concrete strategies, thus increasing individual’s willingness to persevere with them as well. Orlistat use provides a good illustration of this ‘social learning’ effect. Both study arms were offered orlistat in the same way, but greater number in the WAP arm opted to use it. This difference in orlistat use is an intervention effect and so cannot be controlled for. Clients typically react to the offer with uncertainty related to the drug’s side effects. In groups, however, there would usually be someone who had benefited from the drug in the past or who opted for the drug and found it beneficial during the programme, and this encouraged others to try it as well.

Another factor could be the effect of closely monitored concrete tasks that may improve treatment adherence, especially in groups with stress levels and lifestyles not conducive for maintaining difficult behaviour change via self-motivation alone. Encouraging participants to implement for at least one week a range of simple behavioural changes may increase the probability of identifying those that are manageable and beneficial and transfer them into routine lifestyle. The camaraderie that typically develops in WAP groups may represent another possible factor that facilitates attendance and programme adherence.

## Conclusions

In summary, an easy to disseminate public-domain task-based weight management programme can provide effective help to clients across the socioeconomic spectrum and represent value for money to the NHS.

## Additional files

Additional file 1: Monitoring of intervention fidelity. Record of the fidelity of the intervention. (DOCX 14 kb)

Additional file 2: Secondary Outcomes. Secondary outcomes from the study including changes in BMI, waist circumference, proportion of participants losing 5 and 10% of baseline weight and changes in blood pressure. (DOCX 96 kb)

Additional file 3: Statistical Analysis Plan (SAP). Statistical Analysis Plan Version 2.0 14th April 2015. (DOCX 38 kb)

## Abbreviations

BMI: Body Mass Index
CI: Confidence interval
EQ-5D-5 L: European Quality of Life-5 Dimensions-5 Levels
GP: General practitioner
ICER: Incremental cost-effectiveness ratio
IQR: Interquartile range
ISRCTN: International Standard Registered Clinical/soCial sTudy Number
MET: Metabolic equivalent
NHS: National Health Service
NICE: National Institute for Health and Care Excellence
PNI: Practice nurse intervention
QALY: Quality-adjusted life-year
SD: Standard deviation
WAP: Weight Action Programme

## References

[1] Bray GA, Frühbeck G, Ryan DH, Wilding JPH (2016). Management of obesity. Lancet.

[2] Rayner M, Scarborough P (2005). The burden of food related ill health in the UK. J Epidemiol Community Health.

[3] Health and Social Care Information Centre. Statistics on Obesity, Physical Activity and Diet - England, 2015. standard. 2015. http://www.hscic.gov.uk/catalogue/PUB16988. Accessed 23 Jul 2015.

[4] National Center for Health Statistics (US). Health, United States, 2015: With Special Feature on Racial and Ethnic Health Disparities. Hyattsville, MD: National Center for Health Statistics (US); 2016. http://www.ncbi.nlm.nih.gov/books/NBK367640/. Accessed 16 Aug 2016. 27308685

[5] Aveyard P, Lewis A, Tearne S, Hood K, Christian-Brown A, Adab P (2016). Screening and brief intervention for obesity in primary care: a parallel, two-arm, randomised trial. Lancet Lond Engl.

[6] Colquitt JL, Pickett K, Loveman E, Frampton GK. Surgery for weight loss in adults. In: Cochrane Database of Systematic Reviews. John Wiley & Sons, Ltd; 2014. http://onlinelibrary.wiley.com/doi/10.1002/14651858.CD003641.pub4/abstract. Accessed 28 Aug 2016. 10.1002/14651858.CD003641.pub4 PMC9028049 25105982

[7] Yanovski SZ, Yanovski JA (2014). Long-term drug treatment for obesity: a systematic and clinical review. JAMA..

[8] Avenell A, Brown TJ, McGee MA, Campbell MK, Grant AM, Broom J (2004). What interventions should we add to weight reducing diets in adults with obesity? A systematic review of randomized controlled trials of adding drug therapy, exercise, behaviour therapy or combinations of these interventions. J Hum Nutr Diet.

[9] Wadden TA, Volger S, Sarwer DB, Vetter ML, Tsai AG, Berkowitz RI (2011). A two-year randomized trial of obesity treatment in primary care practice. N Engl J Med.

[10] Shaw K, O’Rourke P, Del Mar C, Kenardy J. Psychological interventions for overweight or obesity. Cochrane Database Syst Rev. 2005:CD003818. 10.1002/14651858.CD003818.pub2 15846683

[11] Hartmann-Boyce J, Johns DJ, Jebb SA, Aveyard P (2014). Behavioural weight management review group. Effect of behavioural techniques and delivery mode on effectiveness of weight management: systematic review, meta-analysis and meta-regression. Obes Rev Off J Int Assoc Study Obes.

[12] Hartmann-Boyce J, Johns DJ, Jebb SA, Summerbell C, Aveyard P (2014). Behavioural weight management review group. Behavioural weight management programmes for adults assessed by trials conducted in everyday contexts: systematic review and meta-analysis. Obes Rev.

[13] Magkos F, Fraterrigo G, Yoshino J, Luecking C, Kirbach K, Kelly SC, et al. Effects of moderate and subsequent progressive weight loss on metabolic function and adipose tissue biology in humans with obesity. Cell Metab 2016;0. doi:10.1016/j.cmet.2016.02.005. 10.1016/j.cmet.2016.02.005 PMC4833627 26916363

[14] Harvey JR, Ogden DE (2014). Obesity treatment in disadvantaged population groups: where do we stand and what can we do?. Prev Med.

[15] Jolly K, Lewis A, Beach J, Denley J, Adab P, Deeks JJ (2011). Comparison of range of commercial or primary care led weight reduction programmes with minimal intervention control for weight loss in obesity: lighten up randomised controlled trial. BMJ..

[16] Counterweight Project Team (2008). Evaluation of the counterweight Programme for obesity management in primary care: a starting point for continuous improvement. Br J Gen Pract J R Coll Gen Pract.

[17] The Counterweight Project Team (2004). Current approaches to obesity management in UK primary care: the counterweight Programme. J Hum Nutr Diet.

[18] Team CP, Bell-Higgs AE, Brosnahan NT, Clarke AM, Dow MSA, Haynes SM, et al. The implementation of the Counterweight Programme in Scotland, UK. 2012. https://openair.rgu.ac.uk/handle/10059/810. Accessed 21 Aug 2017. 10.1093/fampra/cmr074 22399544

[19] Hajek P, Humphrey K, McRobbie H (2010). Using group support to complement a task-based weight management programme in multi-ethnic localities of high deprivation. Patient Educ Couns.

[20] English indices of deprivation 2010 - Publications - GOV.UK. https://www.gov.uk/government/statistics/english-indices-of-deprivation-2010. Accessed 31 Oct 2016.

[21] McRobbie H, Hajek P, Peerbux S, Kahan BC, Eldridge S, Trépel D, Parrott S, Griffiths C, Snuggs S, Myers Smith K. Tackling obesity in areas of high social deprivation: clinical effectiveness and cost-effectiveness of a task-based weight management group programme a randomised controlled trial and economic evaluation. Health Technol Assess. 2016;20. 10.3310/hta20790. 10.3310/hta20790 PMC5107886 27802843

[22] NICE. Weight management: lifestyle services for overweight or obese adults. Public health guideline [PH53]. London: National Institute for Health and Care Excellence. https://www.nice.org.uk/guidance/ph53. Accessed 22 Mar 2019.

[23] Healthy food & activity tips for you & your kids | Change4Life. http://www.nhs.uk/change4life/Pages/change-for-life.aspx. Accessed 2 Apr 2016.

[24] NHS. Health A-Z. Obesity - Treatment. https://www.nhs.uk/conditions/obesity/treatment/. Accessed 21 Mar 2019.

[25] Craig CL, Marshall AL, Sjöström M, Bauman AE, Booth ML, Ainsworth BE (2003). International physical activity questionnaire: 12-country reliability and validity. Med Sci Sports Exerc.

[26] White MA, Whisenhunt BL, Williamson DA, Greenway FL, Netemeyer RG (2002). Development and validation of the food-craving inventory. Obes Res.

[27] de Lauzon B, Romon M, Deschamps V, Lafay L, Borys J-M, Karlsson J (2004). The three-factor eating questionnaire-R18 is able to distinguish among different eating patterns in a general population. J Nutr.

[28] EuroQol Group (1990). EuroQol--a new facility for the measurement of health-related quality of life. Health Policy Amst Neth.

[29] White IR, Horton NJ, Carpenter J, Pocock SJ (2011). Strategy for intention to treat analysis in randomised trials with missing outcome data. BMJ..

[30] Kahan BC, Morris TP (2013). Assessing potential sources of clustering in individually randomised trials. BMC Med Res Methodol.

[31] Kahan BC, Morris TP (2012). Improper analysis of trials randomised using stratified blocks or minimisation. Stat Med.

[32] Kahan BC, Jairath V, Doré CJ, Morris TP (2014). The risks and rewards of covariate adjustment in randomized trials: an assessment of 12 outcomes from 8 studies. Trials..

[33] White IR, Thompson SG (2005). Adjusting for partially missing baseline measurements in randomized trials. Stat Med.

[34] NHS. NHS reference costs 2012 to 2013 - Publications - GOV.UK. 2013. https://www.gov.uk/government/publications/nhs-reference-costs-2012-to-2013. Accessed 21 Apr 2014.

[35] Curtis L (2013). Unit costs of health and social care.

[36] Oppe M, Devlin NJ, van Hout B, Krabbe PFM, de Charro F (2014). A program of methodological research to arrive at the new international EQ-5D-5L valuation protocol. Value Health J Int Soc Pharmacoeconomics Outcomes Res.

[37] Wadden TA, Butryn ML, Hong PS, Tsai AG (2014). Behavioral treatment of obesity in patients encountered in primary care settings: a systematic review. JAMA..

[38] Jebb SA, Ahern AL, Olson AD, Aston LM, Holzapfel C, Stoll J (2011). Primary care referral to a commercial provider for weight loss treatment versus standard care: a randomised controlled trial. Lancet.

[39] Aveyard P, Lewis A, Tearne S, Hood K, Christian-Brown A, Adab P, et al. Screening and brief intervention for obesity in primary care: a parallel, two-arm, randomised trial. Lancet. 2016. 10.1016/S0140-6736(16)31893-1. 10.1016/S0140-6736(16)31893-1 PMC5121130 27789061

[40] Neter JE, Stam BE, Kok FJ, Grobbee DE, Geleijnse JM (2003). Influence of weight reduction on blood pressure: a meta-analysis of randomized controlled trials. Hypertension..

[41] Lancaster T, Stead LF (2017). Individual behavioural counselling for smoking cessation. Cochrane Database Syst Rev.

