# Supplementary material for: Randomised controlled trial and economic evaluation of a task-based weight management group programme
Source: BMC Public Health. 2019 Apr 2;19:365. doi: 10.1186/s12889-019-6679-3 (PMC6444848; doi:10.1186/s12889-019-6679-3)
Supplement: Supplementary file 1 — Monitoring of intervention fidelity. Record of the fidelity of the intervention. (DOCX 14 kb) [file 12889_2019_6679_MOESM1_ESM.docx]

**Additional file 1 – Monitoring of intervention fidelity**

## Monitoring of intervention fidelity

For the WAP intervention Hayden McRobbie (HM) attended five sessions led by each advisor (two in the early phase of the trial and then quarterly) and formally checked the conduct of the session against the counselling protocol to provide feedback to the advisors and record fidelity of the intervention. HM attended five sessions led by each advisor in the Nurse intervention (two in the early phase of the trial and then quarterly) and Peter Hajek attended one session with each Nurse advisor. They recorded fidelity of the intervention, which was considered good at all sessions, and provided feedback to the advisors.
