# Supplementary material for: Randomised controlled trial and economic evaluation of a task-based weight management group programme
Source: BMC Public Health. 2019 Apr 2;19:365. doi: 10.1186/s12889-019-6679-3 (PMC6444848; doi:10.1186/s12889-019-6679-3)
Supplement: Supplementary file 2 — Secondary Outcomes. Secondary outcomes from the study including changes in BMI, waist circumference, proportion of participants losing 5 and 10% of baseline weight and changes in blood pressure. (DOCX 96 kb) [file 12889_2019_6679_MOESM2_ESM.docx]

**Additional file 2 – Statistical Analysis Plan**

**A peer-support weight action programme to supplement brief advice in general practice (SWAP)**

**Statistical Analysis Plan**

Version: 2.0

Date: 14^th^ April, 2015

| Person(s) contributing to the analysis plan | |
| --- | --- |
| Name(s) and position(s) | Hayden McRobbie (Chief Investigator)  Brennan Kahan (Statistician)  Sarrah Peerbux (Research Health Psychologist)  Sandra Eldridge (Statistician) |
| Authorisation | |
| Position | **Chief or principal investigator** |
| Name | **Hayden McRobbie** |
| Signature |  |
| Date |  |
| Position | **Senior trial statistician** |
| Name | **Brennan Kahan** |
| Signature |  |
| Date |  |
| Position | **Independent statistician** |
| Name | **Richard Hooper** |
| Tick once reviewed |  |
| Date |  |

# INTRODUCTION

**Purpose of statistical analysis plan**

The purpose of this document is to provide details of the statistical analyses and presentation of results to be reported within the principal paper(s) of the SWAP trial. Subsequent papers of a more exploratory nature (including those involving baseline data only) will not be bound by this strategy but will be expected to follow the broad principles laid down in it. Any exploratory, post hoc or unplanned analyses will be clearly identified in the respective study analysis report.

The structure and content of this document provides sufficient detail to meet the requirements identified by the International Conference on Harmonisation (ICH) and the PCTU SOP (PCTU/07).

**Members of the writing committee**

Brennan Kahan and Hayden McRobbie were primarily responsible for writing the Statistical Analysis Plan, with input from other members of the Trial Management Group.

This document has been finalised before any members of the Trial Management Group had access to the trial data, or were unblinded to trial results.

**Summary**

The SWAP trial aims to determine whether a group-based weight management programme (Weight Action Programme; WAP) targeting underprivileged groups is superior to ‘best practice’ weight management that is provided in primary care by practice nurses.

**Background to the Weight Action Programme**
Weight Action Programme (WAP) is a multi-modal health behaviour modification intervention developed at the Wolfson Institute of Preventive Medicine via extensive client feedback and piloting with underprivileged groups since 2002. The programme is a multi-component service that aims to provide participants with tools to lose weight and maintain a long-term healthy lifestyle. The contents include the standard elements of cognitive behavioural interventions, dietary advice, self-monitoring, information on healthy cooking and eating and caloric content of food, cue management, provision of opportunities for exercise and close monitoring of exercise levels, and a range of concrete and verifiable tasks agreed individually with each participant. Participants are asked to wear a pedometer in order to record daily number of steps at baseline. Throughout the course, individual pedometer step targets are gradually increased until an optimal sustainable level is reached. An innovative feature of the programme consists of the use of group-oriented interventions aiming to increase participant retention, involvement and adherence to weekly tasks. This also makes the programme more cost-effective. The focus of the WAP course is to help participants to maintain a healthy lifestyle after the programme finishes.

The programme has been developed to cater specifically for underprivileged groups including ethnic minorities. Where information is imparted, it is mostly in pictorial and easily understandable format.

WAP has been evaluated in two pilot studies of 162 overweight adults (mean BMI of 35 kg/m2) from multi-ethnic areas of high deprivation.[1] The average weight loss was 2.8kg at end of treatment and 4.5kg at 3-month follow-up (with 24% participants attending follow-up losing 5% or more of their body weight). Limited promotion via GP practices and local adverts generated a large volume of interest. The client retention was at least as good as in comparable programs conducted in research settings with more traditional clients (59% completed the 6-week treatment) and the program received very high approval ratings. Clients also demonstrated significant improvements in knowledge of healthy eating, and in their exercise levels as measured by pedometer monitoring. Clients considered the group support essential in helping them to stick to their tasks and to lose weight.[1] WAP also includes information on orlistat.

We recruited from and conducted the interventions in two GP practices, one in the London borough of Hackney and the other in Tower Hamlets. Both boroughs have a high level of deprivation.

**Changes from planned analysis in the protocol**

In the original trial protocol we specified we would use a baseline-observation-carried-forward approach (BOCF) for dealing with patients with missing weight data during follow-up. This approach assumes that all those who were lost to follow-up returned to their exact baseline weight. Whilst this approach has been commonly used in other randomised controlled trials, it is problematic because it will provide biased estimates of the treatment effect when this assumption is incorrect (i.e. when participants do not return to their exact baseline weight when they fail to show up to their 6 or 12 month appointment). In addition, BOCF will often lead to an inflated type I error (false-positive) rate as it tends to underestimate the standard error for the treatment effect (due to ignoring the within-patient variability in weight when imputing using BOCF).

We have therefore decided to use a mixed-effects linear regression model for the primary analysis. This analysis method provides unbiased estimates of treatment effect and correct type I error rates provided the data is missing-at-random (MAR); that is, that the probability that a participant is lost to follow-up depends on either their previous weight measurements (e.g. their weight at baseline and 6 months if they are lost-to-follow-up at 12 months), and baseline patient characteristics (See section 5 for variables we are adjusting for). [2]

This strategy of analysis has been widely recommended in the presence of missing outcome data. We made the decision to change analysis methods before we had any access to the trial data, or ongoing trial results, and therefore there is no risk of bias associated with this decision.

**Changes from SAP version 1.0**
Version 2.0 of the SAP specifies that all linear mixed-effects models will employ the Kenward-Roger degree-of-freedom correction. This decision was undertaken prior to any member of the trial team having access to unblinded data, or ongoing trial results.

1. **STUDY OBJECTIVES AND ENDPOINTS**

**Study objectives**

Primary objectives

To determine if WAP can generate a better sustained weight loss over 12 months in overweight adults than best-practice intervention that is routinely provided by nurses in general practice.

Secondary objectives

1. To determine the cost-effectiveness (in terms of costs of interventions and QALYs derived from the EQ-5D) of the two interventions

**Outcome measures**

Primary outcomes

The primary outcome measure is the change in weight (in kg) at 12 months post-randomisation.

Secondary outcomes

- Change in weight (in kg) at 1, 2, and 6 months post-randomisation.
- Change in BMI at 1, 2, 6 and 12 months post-randomisation. BMI is calculated as weight (in kg) divided by the square of height (in metres). The height measured at screening will be used for each follow-up assessment.
- Change in waist circumference (in cm) at 2, 6 and 12 months post-randomisation.
- Change in systolic blood pressure (mmHg) at 2, 6 and 12 months post-randomisation.
- Change in diastolic blood pressure (mmHg) at 2, 6 and 12 months post-randomisation.
- Change in the Food Craving Inventory score (Frequency domain) at 1, 2, 6, and 12 months post-randomisation.
- Change in the Food Craving Inventory score (Strength domain) at 1, 2, 6, and 12 months post-randomisation.
- Change in Food Knowledge Assessment Questionnaire score at 2, 6, and 12 months post-randomisation.
- Change in the Three Factor Eating Questionnaire score (Cognitive Restraint domain) at 2, 6, and 12 months post-randomisation.
- Change in the Three Factor Eating Questionnaire score (Uncontrolled Eating domain) at 2, 6, and 12 months post-randomisation.
- Change in the Three Factor Eating Questionnaire score (Emotional Eating domain) at 2, 6, and 12 months post-randomisation.
- Change in the International Physical Activity Questionnaire (IPAQ) score (MET-minutes/week domain) at 2, 6, and 12 months post-randomisation.
- Change in the International Physical Activity Questionnaire (IPAQ) score (Sitting domain) at 2, 6, and 12 months post-randomisation.
- Proportion of participants losing 5% of body weight at 2, 6, and 12 months post-randomisation.
- Proportion of participants losing 10% of body weight at 2, 6, and 12 months post-randomisation.

Scoring details for the Food Craving Inventory, the Food Knowledge Assessment Questionnaire, the Three Factor Eating Questionnaire, and the International Physical Activity Questionnaire are available in Appendix 2.

Weight, BMI, waist and blood pressure outcomes were measured by researchers who were blind to treatment arm. These researchers were affiliated with the trial team, but were involved only in collecting outcomes during follow-up, and had no role in providing the intervention, and no contact with patients other than whilst collecting follow-up measurements.

1. **STUDY METHODS**

**Overall study design and plan**

Target for randomisation: 220 intervention and 110 control participants

Date of first randomisation: 27/09/2012

Date of last randomisation: 30/01/2014

Trial design: Individually randomised, parallel group

Who is blinded: Researchers affiliated with the study team conducting measurements at 6 and 12-month follow-up. Patients and those delivering the intervention are aware of the patient’s treatment allocation.

Randomised Interventions: Intervention (WAP) vs. control (Nurse counselling)

Allocation ratio: 2:1

**Selection of study population**

The study population was selected from people responding to letters and text messages sent from their GP surgery, posters in surgery waiting areas, direct referrals from GP staff and advertisements in local papers.

Participants were eligible to take part if they were age 18 years and older, wanted to lose weight, and had a BMI of 30 kg/m^2^ or over, or a BMI of 28 kg/m^2^ or over with co-morbidities.

Participants were excluded from participating if they could not read, write, or speak English, had a BMI over 45 kg/m2, had lost more than 5% of their body weight in the previous 6 months, were pregnant, currently taking psychiatric medications, were not registered with a GP, or currently involved in another research project.

**Method of treatment assignment and randomisation**

Participants were randomly allocated to the two treatment arms in a 2:1 ratio (intervention:control) by means of an independent web-based randomisation service. Allocation was via random permuted blocks stratified by GP Practice (Lawson vs. Barkantine) with randomly varying block sizes of 18, 21, and 24.

Randomisation was undertaken within each GP practice. Study staff accessed the web-based randomisation programme developed by the Sheffield Clinical Trials Unit, University of Sheffield and entered the participant ID number into the programme. No other information was entered. The allocation was immediately provided by the programme and participants were given instruction on what to do for the next sessions. Neither participant nor study staff were blind to the allocation after this point.

**Treatment masking (Blinding)**

Participants and study staff providing the interventions were not blinded. However the study staff collecting the measurements at 6 and 12-month follow-up (the primary endpoint) were blinded to allocation.

The statistician (and all other staff who have access to outcome data) remained blinded until the database was finalised and Statistical Analysis Plan is signed off.

**Sample size determination**

A clinically significant effect can be achieved with 3-5 kg weight loss in obese people.[3] We assumed that WAP would increase weight loss by 2.6kg compared with usual care (WAP 3kg vs. usual care 0.4kg) for participants available for follow-up at one year, and that there would be no difference in weight loss between treatment groups for participants not available for follow-up. Assuming that 50% of participants in both treatment groups were available for follow-up at one year, the difference in weight loss between groups would be 1.3kg (WAP 1.5kg vs. usual care 0.2kg). Assuming a standard deviation of 3 in both treatment groups, and a 5% two-sided significance level, we would require 112 participants in each group to detect this mean difference with 90% power. Our estimate of 50% loss to follow-up is conservative and based on international experience in this field and existing data from similar underprivileged and highly mobile populations and interventions.

To account for potential clustering effects due to group treatment in the intervention arm, assuming a mean cluster size of 18 and an intra-cluster correlation coefficient of 0.05, a total of 208 individuals will be required in the intervention arm. The same power can be achieved with 108 in the control arm and 216 in the intervention arm which we have increased to 110 in the control arm and 220 in the intervention arm to give an allocation ratio between the two arms (2:1) which can be expressed in whole numbers. Thus we require a total of 330 individuals for the entire study

1. **DATA COLLECTION**

**Baseline**

The following variables were collected at baseline

- ***Demographics:*** includes age, sex, ethnicity, employment, level of education
- ***Health and lifestyle:*** includes smoking status, alcohol consumption, and general health
- ***Weight loss history:*** includes number of past weight-loss attempts, methods used, most weight ever lost, and regular monitoring of weight.
- ***Concurrent medications:*** all current medications are recorded.
- ***Height and weight:*** measured in centimeters and kilograms. BMI calculated from these.
- ***Waist circumference:*** measured in centimetres.
- ***Blood pressure:*** resting blood pressure recorded.

The following validated questionnaires are also administered at baseline:

- International Physical Activity Questionnaire [4]
- Food knowledge assessment
- Food craving inventory [5]
- Three Factor Eating Questionnaire [6]
- EQ-5D
- Use of health services questionnaire

**Follow up**

The following variables were collected during follow-up visits: weight, waist circumference, blood pressure, International Physical Activity Questionnaire [4], Food knowledge assessment, Food craving inventory, Three Factor Eating Questionnaire [6], EQ-5D, use of health services questionnaire, adverse events, participant feedback and medication use.

In the intervention arm the following were collected during the 8-week intervention phase: pedometer use, food diary use, and adherence to weekly tasks (e.g. increase fruit and vegetable intake, increase exercise, monitoring television and computer use).

**Timing of data collection**

The recruitment period was: September 2012– January 2014 (17 months) and the study sessions were conducted as follows:

Week -1: Screening

Week 0: Randomisation

Weeks 1-8: Intervention group – 8 weekly sessions

Control group – 4 fortnightly sessions

Months 3-12: Intervention group – 10 monthly follow-up sessions

Control group – 6 and 12 month follow-up sessions only

**Database**

Description

Data were entered into the online database, ‘Oracle Database version 11’, hosted at the Barts Cancer Centre. The Electronic Data Capture forms are web based and built using Java with data validation in JavaScript (Java framework Struts 2).

Data quality

When recruitment and follow-up are complete, the study team will clean the data in the following way: values for each variable will be sorted, and those at the extremes will be checked to ensure that they are within the expected range.

Source data verification will also be conducted: a random sample of 10% of CRFs will be selected, and a member of QA team (PCTU) will compare all written entries with those entered onto the main study database. The pre-specified data quality target is ≤ 2% discrepancy rate between entries in the CRF and the electronic database. If an error is found in >2% of entries, the quality target for data entry will not have been met, and all CRF data will be cross-checked against data in the study database. (This would be done by counting up the maximum number of data items that could be entered for a patient on each of the CRFs, ignoring free text fields. Errors will be tallied and these would include any items that were inadvertently missed out.)

Derived and computed variables

All derived and computed variables will be documented in the analysis programmes.

1. **GENERAL ISSUES FOR STATISTICAL ANALYSIS**

**General analysis principles**

The main analysis for each outcome will use intention-to-treat (ITT) principles, meaning that all participants with a recorded outcome will be included in the analysis, and will be analysed according to the treatment group to which they were randomised. More information on which participants will be included in each analysis is available in the section below. All p-values will be two sided, and the significance level is set at 5%.

Analyses for all outcomes will be presented as:

- The number of participants included in the analysis, by treatment group;
- A summary measure of the outcome, by treatment group (e.g. mean (SD) for continuous outcomes, number (%) for binary outcomes);
- A treatment effect, with a 95% confidence interval;
- A two-sided p-value.

All analyses will account for clustering by group in the intervention arm, and clustering by nurse in the control arm. Each patient will be defined as belonging to a cluster, defined by which group they belonged to if they were in the intervention arm, and which nurse they were treated by if they were in the control arm. This variable will be included as a random intercept in a mixed-effects regression model. This analysis assumes the intraclass correlation coefficient is the same between groups in the intervention arm as it is between nurses in the control arm. The Kenward-Roger degree-of-freedom correction will be employed for all linear mixed-effects models.

All analyses will adjust for baseline weight, age, gender, ethnicity (White British, White other, Black, Asian, Mixed, or other), smoking status (smoker vs. non-smoker) and GP practice (Lawson vs. Barkantine) as covariates in a regression model. Outcomes which are measured at baseline will also be adjusted for the value of the outcome at baseline (this includes weight, BMI, waist circumference, systolic and diastolic blood pressure, Food Craving Inventory, Food Knowledge Assessment, Three Factor Eating Questionnaire, and IPAQ). Continuous covariates (baseline weight, age) will be assumed to have a linear association with outcome. Binary and categorical covariates (gender, ethnicity, smoking status, and GP practice) will be included in the regression model using indicator (dummy) variables. Missing baseline data will be accounted for using mean imputation.

**Missing data for outcomes**

For outcomes that are measured at multiple time points during follow-up, we have based our analysis strategy on that proposed by White et al 2011[7]. To deal with incomplete data (i.e. when patients have missing data at one of the follow-up time points) we will:

1. Attempt to follow up all randomised patients even if they withdraw from the study
2. Perform a main analysis of all observed data that are valid under a plausible assumption about the missing data
3. Perform sensitivity analyses to explore the effect of departures from the assumptions made in the main analysis
4. Account for all randomised participants, at least in the sensitivity analyses

We will therefore (a) include all patients with at least one post-randomisation assessment (i.e. if they have recorded data for at least one follow-up time point) in the analysis; (b) use mixed-effects models adjusted for baseline covariates, which assumes that the data are missing-at-random (i.e. they are missing based on their observed outcome at other time-points, and other patient characteristics); and (c) perform sensitivity analyses under other missing data assumptions (e.g. that patients who were lost-to-follow-up gained more weight than patients who remained in the trial).

**Analysis of primary outcome**

The primary outcome (change in weight at 12 months post-randomisation) will be analysed using a mixed-effects linear regression model. The model will include change in weight at 1, 2, 6 and 12 months as outcomes.

The model will include a random intercept for ‘cluster’ (group or nurse, depending on treatment arm). The correlation between observations at different time points from the same patient (1, 2, 6, and 12 months) will be modelled using an unstructured correlation structure. The model will be estimated using restricted maximum likelihood (REML). Treatment arm, time point (month 1, 2, 6, or 12), and the interaction between treatment arm and time point will be included in the model as fixed factors. Time point will be included as an indicator variable. The covariates listed in section 5 will also be included in the model as fixed factors.

The analysis will be implemented in Stata as follows:

mixed outcome treatment##time covariates || cluster_id:, || ///

patient_id:, noconstant residuals(unstructured, t(time)) stddev reml dfmethod(kroger)

If this model fails to converge, we will run the model again using the correlation structure

*residuals(ar 2 , t(time))*. If the model still fails to converge, we will use *residuals(ar 1 , t(time))*.

**Sensitivity analyses for primary outcome**

#### *Missing data*

#### We will perform two sensitivity analyses to assess the robustness of our primary analysis to different assumptions regarding the missing data. These sensitivity analyses will be performed for the primary outcome (change in weight at 12 months).

#### A complete case analysis, where only patients with recorded data at 12 months are included

- An analysis which assumes data missing at 12 months is missing-not-at-random.

We will perform the second sensitivity analysis (where data missing at 12 months is assumed to be missing-not-at-random) using the formula ∆ = ∆_CC_ + Y_1_P_1_ – Y_2_P_2_, where ∆ is the treatment effect under the missing-not-at-random scenario, ∆_CC_ is the treatment effect from a complete case analysis, Y_1_ and Y_2_ are the assumed mean responses for participants with missing data in treatment groups 1 and 2 respectively, P_1_ and P_2_ are the proportion of participants who were excluded from the analysis in groups 1 and 2 respectively, and groups 1 and 2 represent the intervention and control groups respectively. The standard error for ∆ is assumed to be approximately equal to the standard error for ∆_CC._  Y_2_ will be varied between -10, -5, -2.5, 0, 2.5, 5, and 10. Negative values indicate the participant lost weight at 12 months, positive values indicate they gained weight, and a value of 0 indicates there was no change from baseline. For each value of Y_2_, Y_1_ will be set to Y_2_ - 5, Y_2_, and Y_2_ + 5.

For example, for Y_2_ = 10, this would indicate an assumption that patients in treatment arm 2 (the control arm) who were lost to follow-up at 12 months, had gained 10kg on average at 12 months. Y_1_ would vary between 5, 10, and 15, indicating the assumption that patients in treatment arm 1 (the intervention arm) who were lost to follow-up had gained 5kg on average at 12 months (5kg less than those in the control arm), 10kg (the same amount as those in the control arm), or 15kg (5kg more than those in the control arm).

***Patients who became pregnant or had bariatric surgery during follow-up***

We will perform a sensitivity analysis to assess the impact of patients who became pregnant or underwent bariatric surgery during follow-up on results. This analysis will be performed for the primary outcome. This sensitivity analysis will involve including only weight measurements collected prior to pregnancy/bariatric surgery in the analysis; weight measurements collected after pregnancy/bariatric surgery will be set to missing. This analysis will be performed using the same methods as for the primary analysis.

**Analysis of secondary outcomes**

*Change in weight at 1, 2, and 6 months*

This outcome will be included in the same model as the primary outcome.

*Change in BMI at 1, 2, 6 and 12 months*

These outcomes will be analysed using the same method as change in weight at 6 and 12 months, with the exception that baseline BMI will be included as a covariate in the regression model, as opposed to baseline weight. BMI measurements at 1, 2, 6, and 12 months will be included in the model.

*Change in waist circumference at 2, 6 and 12 months*

These outcomes will be analysed using the same method as change in weight at 6 and 12 months with the exception that baseline waist circumference will be included as a covariate in the regression model, as opposed to baseline weight. Waist circumference measurements at 2, 6, and 12 months will be included in the model.

*Change in systolic blood pressure at 2, 6 and 12 months*

These outcomes will be analysed using the same method as change in weight at 6 and 12 months. The baseline value of systolic blood pressure will also be included as a covariate in the model. Systolic blood pressure measurements at 2, 6, and 12 months will be included in the model.

*Change in diastolic blood pressure at 2, 6 and 12 months*

These outcomes will be analysed using the same method as change in weight at 6 and 12 months. The baseline value of diastolic blood pressure will also be included as a covariate in the model. Diastolic blood pressure measurements at 2, 6, and 12 months will be included in the model.

*Change in Food Craving Inventory (frequency domain) at 1, 2, 6 and 12 months*

These outcomes will be analysed using the same method as change in weight at 6 and 12 months. The baseline value of the Food Craving Inventory (frequency domain) will also be included as a covariate in the model. Frequency domain measurements at 2, 6, and 12 months will be included in the model.

Treatment effect estimates will only be presented at 6 and 12 months; data from month 2 is included in the model to increase power, and to make the missing-at-random assumption more plausible.

*Change in Food Craving Inventory (strength domain) at 1, 2, 6 and 12 months*

These outcomes will be analysed using the same method as change in weight at 6 and 12 months. The baseline value of the Food Craving Inventory (frequency domain) will also be included as a covariate in the model. Strength domain measurements at 2, 6, and 12 months will be included in the model.

*Change in Food Knowledge Assessment at 2, 6 and 12 months*

These outcomes will be analysed using the same method as change in weight at 6 and 12 months. The baseline value of food knowledge will also be as a covariate in the model. Food Knowledge Assessment measurements at 2, 6, and 12 months will be included in the model.

*Change in Three Factor Eating Questionnaire (Cognitive Restraint domain) at 2, 6 and 12 months*

These outcomes will be analysed using the same method as change in weight at 6 and 12 months. The baseline value of the Three Factor Eating Questionnaire (Cognitive Restraint domain) will also be included as a covariate in the model. Cognitive Restraint domain measurements at 2, 6, and 12 months will be included in the model.

*Change in Three Factor Eating Questionnaire (Uncontrolled Eating domain) at 2, 6 and 12 months*

These outcomes will be analysed using the same method as change in weight at 6 and 12 months. The baseline value of the Three Factor Eating Questionnaire (Uncontrolled Eating domain) will also be included as a covariate in the model. Uncontrolled Eating domain measurements at 2, 6, and 12 months will be included in the model.

*Change in Three Factor Eating Questionnaire (Emotional Eating domain) at 2, 6 and 12 months*

These outcomes will be analysed using the same method as change in weight at 6 and 12 months. The baseline value of the Three Factor Eating Questionnaire (Emotional Eating domain) will also be included as a covariate in the model. Emotional Eating domain measurements at 2, 6, and 12 months will be included in the model.

*Change in International Physical Activity Questionnaire (MET-minutes/week domain) at 2, 6 and 12 months*

These outcomes will be analysed using the same method as change in weight at 6 and 12 months. The baseline value of the MET-minutes/week domain will also be included as a covariate in the model. MET-minutes/week domain measurements at 2, 6, and 12 months will be included in the model.

*Change in International Physical Activity Questionnaire (sitting domain) at 2, 6 and 12 months*

These outcomes will be analysed using the same method as change in weight at 6 and 12 months. The baseline value of the sitting domain will also be included as a covariate in the model. Sitting domain measurements at 2, 6, and 12 months will be included in the model.

*Proportion of participants losing 5% of body weight at 2, 6, and 12 months*

These outcomes will be analysed using a mixed-effects logistic regression model. The model will include whether the participant had lost 5% of their body weight at 2, 6 and 12 months as outcomes.

The model will include three levels: the top level will include a random intercept for ‘cluster’ (group or nurse, depending on treatment arm). The second level will include a random intercept for patient, and a random slope for time point. Treatment arm, time point, and the interaction between treatment arm and time point will be included in the model as fixed factors. Time point will be included as an indicator variable. The covariates listed in section 5 will also be included in the model as fixed factors.

The analysis will be implemented in Stata as follows:

meqrlogit outcome treatment##time covariates || cluster_id:, || ///

patient_id: time, cov(exch)

If this model fails to converge, we will run the model again after removing the random slope for time at the second level.

*Proportion of participants losing 10% of body weight at 2, 6, and 12 months*

These outcomes will be analysed using the same methods as the proportion of participants losing 5% of body weight at 2, 6, and 12 months.

**Subgroup analyses**

No subgroup analyses will be performed.

**Other data summaries**

- Number of participants on both treatment arms who began taking orlistat during follow-up
- Compare weight change at 12 months in participants who received orlistat during follow-up vs. those who did not
- Summary measures for the feedback questionnaire form (mean and SD, number and percent) in both treatment arms for Q1, Q2, and Q4

1. **Figures**

**Participant flow**

Participant throughput will be summarized in a CONSORT diagram (see figure 1).

| **Figure 1: Consort Diagram** | | | | | | | | | | | |
| --- | --- | --- | --- | --- | --- | --- | --- | --- | --- | --- | --- |
|  |  |  |  | Screened for eligibility  (N= XX) | | | |  |  |  |  |
|  |  |  |  |  |  |  |  |  |  |  |  |
|  |  |  |  |  |  |  |  | Excluded (N=XX)   - Not eligible (N=XX) - Declined to participate (N=XX) | | | |
|  |  |  |  |  |  |  |  |  |  |  |  |
|  |  |  |  | Randomised (N=XX) | | | |  |  |  |  |
|  |  |  |  |  |  |  |  |  |  |  |  |
| Allocated to Intervention (WAP) (N=XX)   - Began receiving treatment (N=XX) - Completed at least 50% of sessions (N=XX) | | | |  |  |  |  | Allocated to Control (nurse advice) (N=XX)   - Began receiving treatment (N=XX) - Completed at least 50% of sessions (N=XX) | | | |
|  |  |  |  |  |  |  |  |  |  |  |  |
| Attended 8-week follow-up (N=XX) | | | |  |  |  |  | Attended 8-week follow-up (N=XX) | | | |
|  |  |  |  |  |  |  |  |  |  |  |  |
| Attended 6-month follow-up (N=XX) | | | |  |  |  |  | Attended 6-month follow-up (N=XX) | | | |
|  |  |  |  |  |  |  |  |  |  |  |  |
| Attended 12-month follow-up (N=XX)   - Dropped out prior to the end of the study (N=XX) | | | |  |  |  |  | Attended 12-month follow-up (N=XX)   - Dropped out prior to the end of the study (N=XX) | | | |
|  |  |  |  |  |  |  |  |  |  |  |  |
| - Patients included in the analysis for the primary outcome (N=XX) | | | |  |  |  |  | - Patients included in the analysis for the primary outcome (N=XX) | | | |
|  |  |  |  |  |  |  |  |  |  |  |  |

**Other figures**

For certain outcomes, we will produce two graphs. The first graph will show the mean outcome within each treatment group (i.e. the mean outcome in the intervention arm, and the mean outcome in the control arm) at each time-point of follow-up. The mean outcome at each time point will be presented with a 95% confidence interval. The second graph will show the estimated treatment effect (with a 95% CI) at each time point.

These graphs will be produced for the following outcomes:

- Change in weight at 1, 2, 6, and 12 months
- Change in BMI at 1, 2, 6, and 12 months
- Change in waist circumference at 2, 6, and 12 months
- Change in systolic blood pressure at 2, 6, and 12 months

1. **Tables**

**Table 1 - Baseline measurements**

|  | Usual care (n=…) | WAP (n=…) |
| --- | --- | --- |
| Weight (kg) – mean (SD) |  |  |
| BMI – mean (SD) |  |  |
| Waist circumference – mean (SD) |  |  |
| Systolic blood pressure – mean (SD) |  |  |
| Diastolic blood pressure – mean (SD) |  |  |
| Age (years) – mean (SD) |  |  |
| Female – no. (%) |  |  |
| Food Craving Inventory score – mean (SD) |  |  |
| Frequency domain |  |  |
| Strength domain |  |  |
| Food Knowledge Assessment Questionnaire score – mean (SD) |  |  |
| Three Factor Eating Questionnaire score – mean (SD) |  |  |
| Cognitive Restraint domain |  |  |
| Uncontrolled Eating domain |  |  |
| Emotional Eating domain |  |  |
| International Physical Activity Questionnaire – mean (SD) |  |  |
| MET-minutes/week domain |  |  |
| Sitting domain |  |  |
| Centre – no. (%) |  |  |
| Lawson |  |  |
| Barkantine |  |  |
| Marital status – no. (%) |  |  |
| Single |  |  |
| Separated or divorced |  |  |
| Married or living with partner |  |  |
| Other |  |  |
| Ethnicity – no. (%) |  |  |
| White British |  |  |
| White other |  |  |
| Black |  |  |
| Asian |  |  |
| Mixed |  |  |
| Other |  |  |
| Educational qualification – no. (%) |  |  |
| None |  |  |
| GCSE or equivalent |  |  |
| A-Level or equivalent |  |  |
| Degree or equivalent |  |  |
| Other |  |  |
| Employment status – no. (%) |  |  |
| In paid employment |  |  |
| Unemployed |  |  |
| Looking after the home |  |  |
| Retired |  |  |
| Full time student |  |  |
| Other |  |  |
| Entitled to free prescriptions – no. (%) |  |  |
| Smoking status – no. (%) |  |  |
| Smoker |  |  |
| Non-smoker |  |  |
| Units of alcohol consumed per week – mean (SD) |  |  |
| Family history of being overweight or obese – no. (%) |  |  |
| Mother |  |  |
| Father |  |  |
| Themselves |  |  |
| Number of previous attempts at weight loss – median (IQR) |  |  |
| Greatest previous amount of weight loss – median (IQR) |  |  |

**Table 2 – Characteristics of intervention groups and patient adherence**

|  | Usual care (n=…) | WAP (n=…) |
| --- | --- | --- |
| Number of intervention groups or nurses (usual care) |  |  |
| Number of participants per group – median (IQR) |  |  |
| Number of sessions attended per participant – median (IQR) |  |  |
| Attended more than half the sessions – no. (%) |  |  |

**Table 3 – Number (%) of participants included in the analysis for each outcome**

|  | Usual care (n=…) | WAP (n=…) |
| --- | --- | --- |
| Change in weight |  |  |
| Change in BMI |  |  |
| Change in waist circumference |  |  |
| Change in systolic blood pressure |  |  |
| Change in diastolic blood pressure |  |  |
| Food Craving Inventory score |  |  |
| Frequency domain |  |  |
| Strength domain |  |  |
| Food Knowledge Assessment Questionnaire score |  |  |
| Three Factor Eating Questionnaire score |  |  |
| Cognitive Restraint domain |  |  |
| Uncontrolled Eating domain |  |  |
| Emotional Eating domain |  |  |
| International Physical Activity Questionnaire |  |  |
| MET-minutes/week domain |  |  |
| Sitting domain |  |  |
| Participants losing 5% of their body weight |  |  |
| Participants losing 10% of their body weight |  |  |

**Table 4 – Results for primary and secondary outcomes**

|  | **Usual care (n=…)** | **WAP (n=…)** | **Treatment effect* (95% CI)** | **P-value** |
| --- | --- | --- | --- | --- |
| Change in weight (kg) – mean (SD) |  |  |  |  |
| 1 month |  |  |  |  |
| 2 months |  |  |  |  |
| 6 months |  |  |  |  |
| 12 months |  |  |  |  |
| Change in BMI – mean (SD) |  |  |  |  |
| 1 month |  |  |  |  |
| 2 months |  |  |  |  |
| 6 months |  |  |  |  |
| 12 months |  |  |  |  |
| Change in waist circumference (cm) – mean (SD) |  |  |  |  |
| 2 months |  |  |  |  |
| 6 months |  |  |  |  |
| 12 months |  |  |  |  |
| Change in systolic blood pressure – mean (SD) |  |  |  |  |
| 2 months |  |  |  |  |
| 6 months |  |  |  |  |
| 12 months |  |  |  |  |
| Change in diastolic blood pressure – mean (SD) |  |  |  |  |
| 2 months |  |  |  |  |
| 6 months |  |  |  |  |
| 12 months |  |  |  |  |
| Change in Food Craving Inventory score (Frequency domain) – mean (SD) |  |  |  |  |
| 1 month |  |  |  |  |
| 2 months |  |  |  |  |
| 6 months |  |  |  |  |
| 12 months |  |  |  |  |
| Change in Food Craving Inventory score (Strength domain) – mean (SD) |  |  |  |  |
| 1 month |  |  |  |  |
| 2 months |  |  |  |  |
| 6 months |  |  |  |  |
| 12 months |  |  |  |  |
| Change in Food Knowledge Assessment Questionnaire score – mean (SD) |  |  |  |  |
| 2 months |  |  |  |  |
| 6 months |  |  |  |  |
| 12 months |  |  |  |  |
| Change in Three Factor Eating Questionnaire score (Cognitive Restraint domain) – mean (SD) |  |  |  |  |
| 2 months |  |  |  |  |
| 6 months |  |  |  |  |
| 12 months |  |  |  |  |
| Change in Three Factor Eating Questionnaire score (Uncontrolled Eating domain) – mean (SD) |  |  |  |  |
| 2 months |  |  |  |  |
| 6 months |  |  |  |  |
| 12 months |  |  |  |  |
| Change in Three Factor Eating Questionnaire score (Emotional Eating domain) – mean (SD) |  |  |  |  |
| 2 months |  |  |  |  |
| 6 months |  |  |  |  |
| 12 months |  |  |  |  |
| Change in International Physical Activity Questionnaire (MET-minutes/week domain) – mean (SD) |  |  |  |  |
| 2 months |  |  |  |  |
| 6 months |  |  |  |  |
| 12 months |  |  |  |  |
| Change in International Physical Activity Questionnaire (Sitting domain) – mean (SD) |  |  |  |  |
| 2 months |  |  |  |  |
| 6 months |  |  |  |  |
| 12 months |  |  |  |  |
| Participants losing 5% of their body weight – no. (%) |  |  |  |  |
| 2 months |  |  |  |  |
| 6 months |  |  |  |  |
| 12 months |  |  |  |  |
| Participants losing 10% of their body weight – no. (%) |  |  |  |  |
| 2 months |  |  |  |  |
| 6 months |  |  |  |  |
| 12 months |  |  |  |  |

*Treatment effects are presented as a difference in means (estimated from a mixed-effects regression model) between the two groups (WAP vs. control) for all outcomes apart for the number of participants who lost 5% or 10% of their body weight, where the treatment effect is presented as an odds ratio.

**Table 5 – ICC values for group or nurse at 6 and 12 months**

|  | 6 months | 12 months |
| --- | --- | --- |
| Change in weight |  |  |
| Change in BMI |  |  |
| Change in waist circumference |  |  |
| Change in systolic blood pressure |  |  |
| Change in diastolic blood pressure |  |  |
| Food Craving Inventory score |  |  |
| Frequency domain |  |  |
| Strength domain |  |  |
| Food Knowledge Assessment Questionnaire score |  |  |
| Three Factor Eating Questionnaire score |  |  |
| Cognitive Restraint domain |  |  |
| Uncontrolled Eating domain |  |  |
| Emotional Eating domain |  |  |
| International Physical Activity Questionnaire |  |  |
| MET-minutes/week domain |  |  |
| Sitting domain |  |  |
| Participants losing 5% of their body weight |  |  |
| Participants losing 10% of their body weight |  |  |

**Table 6 – No. (%) of participants in the WAP group using different process measures at each session**

| Process measure | S1 (n=…) | S2 (n=…) | S3 (n=…) | S4 (n=…) | S5 (n=…) | S6 (n=…) | S7 (n=…) | S8 (n=…) |
| --- | --- | --- | --- | --- | --- | --- | --- | --- |
| Pedometer use | No. (%) | No. (%) | No. (%) | No. (%) | No. (%) | No. (%) | No. (%) | No. (%) |
| TV/screen time use | No. (%) | No. (%) | - | - | - | - | - | - |
| Food diary use | No. (%) | - | - | - | - | - | - | - |
| Counted calories | - | No. (%) | - | - | - | - | - | - |
| 5/day | - | - | No. (%) | No. (%) | No. (%) | No. (%) | No. (%) | No. (%) |
| Exercise | - | - | - | No. (%) | No. (%) | No. (%) | No. (%) | No. (%) |
| No junk | - | - | - | - | No. (%) | No. (%) | No. (%) | No. (%) |
| Scales | - | - | - | - | No. (%) | No. (%) | No. (%) | No. (%) |
| Removed triggers | - | - | - | - | - | - | No. (%) | - |

1. **REFERENCES**

1. Hajek P, Humphrey K, McRobbie H: **Using group support to complement a task-based weight management programme in multi-ethnic localities of high deprivation**. *Patient Educ Couns* 2010, **80**:135–137.

2. Wood AM, White IR, Hillsdon M, Carpenter J: **Comparison of imputation and modelling methods in the analysis of a physical activity trial with missing outcomes**. *Int J Epidemiol* 2005, **34**:89–99.

3. Yanovski SZ, Bain RP, Williamson DF: **Report of a National Institutes of Health--Centers for Disease Control and Prevention workshop on the feasibility of conducting a randomized clinical trial to estimate the long-term health effects of intentional weight loss in obese persons**. *Am J Clin Nutr* 1999, **69**:366–372.

4. Craig CL, Marshall AL, Sjöström M, Bauman AE, Booth ML, Ainsworth BE, Pratt M, Ekelund U, Yngve A, Sallis JF, Oja P: **International physical activity questionnaire: 12-country reliability and validity**. *Med Sci Sports Exerc* 2003, **35**:1381–1395.

5. White MA, Whisenhunt BL, Williamson DA, Greenway FL, Netemeyer RG: **Development and validation of the food-craving inventory**. *Obes Res* 2002, **10**:107–114.

6. De Lauzon B, Romon M, Deschamps V, Lafay L, Borys J-M, Karlsson J, Ducimetière P, Charles MA, Fleurbaix Laventie Ville Sante Study Group: **The Three-Factor Eating Questionnaire-R18 is able to distinguish among different eating patterns in a general population**. *J Nutr* 2004, **134**:2372–2380.

7. White IR, Horton NJ, Carpenter J, Pocock SJ: **Strategy for intention to treat analysis in randomised trials with missing outcome data**. *BMJ* 2011, **342**:d40.

**Appendix 1**

***Timing of data collection***

| **Source of data** | **Data collected** |
| --- | --- |
| Baseline questionnaire | Age  Sex  Marital status  Ethnicity  Educational qualification  Employment status  Entitlement to free prescriptions  Smoking status  Alcohol Consumption  Eating Habits  Weight Loss history  Concurrent illnesses/ medications |
| CRF – screening session | Weight (kg)  Height (cm)  BMI  Eligibility checked against inclusion criteria |
| CFR – randomisation session | Weight (kg)  Waist circumference (cm)  Blood pressure  MPSS  Motivation scale  Use of other weight loss methods |
| CRF – control group treatment sessions | Weight (kg)  Waist circumference (cm) (Session 4 only)  Blood pressure (Session 4 only)  MPSS  Motivation scale (Session 1 only)  Use of other weight loss methods  AEs |
| CRF – intervention group treatment sessions | Weight (kg)  Waist circumference (cm) (Session 8 only)  Blood pressure (Session 8 only)  MPSS  Motivation scale (Session 1 only)  Use of other weight loss methods  AEs |
| Task cards (intervention group only) | Pedometer readings (reported and actual)  Step target  Screen time  Completion of food diary  Calorie counting  5/day  Exercise  ‘Said no’ to unnecessary food  Self monitoring on weighing scales |
| CRF – 6 month follow-up | Weight (kg)  Waist circumference (cm)  Blood pressure  Concurrent illness/medications  MPSS  Use of other weight loss methods  AEs |
| CRF – 12 month follow-up | Weight (kg)  Waist circumference (cm)  Blood pressure  Concurrent illness/medications  MPSS  Use of other weight loss methods  AEs |

**Appendix 2**

***Scoring of questionnaires***

**Food Knowledge Assessment score**

The Food Knowledge Assessment score is scored on an 11 point scale (range 0-10), with higher scores indicating more knowledge. It contains 10 questions, and each question is score either 0 or 1. The overall score is calculated by summing the scores of the individual questions.

The scores for the individual questions is shown in the table below. Each question has four possible answers (a, b, c, d); the table indicates which of the four answers results in a score of 1 (all other answers result in a score of 0).

|  | Score=1 if answer is |
| --- | --- |
| Q1 | A |
| Q2 | A |
| Q3 | C |
| Q4 | B |
| Q5 | D |
| Q6 | B |
| Q7 | C |
| Q8 | B |
| Q9 | B |
| Q10 | A |

**Food Craving Inventory score**

Each of the five food types (fatty foods, carbohydrates and starches, sweet foods, savoury snacks, and fruit) is assigned a score from 0 to 5 on both frequency and urge of craving. The frequency domain is then calculated by summing the scores of the individual questions related to frequency; the strength domain is calculated in a similar manner. The overall scores from both domains range from 0 to 25, with higher scores indicating more frequent or stronger urges.

**International Physical Activity Questionnaire**

***MET-minutes/week domain***

This score represents the total MET-minutes/week, and is expressed on a continuous scale with a minimum score of 0. It is calculated as:

MET-minutes/week = 3.3*(walking intensity minutes)*(walking intensity days) +

4.0* (moderate intensity minutes)*(moderate intensity days) +

8.0* (vigorous intensity minutes)*( vigorous intensity days)

***Sitting domain***

This score represents the number of minutes per day spent sitting. It is calculated directly from question 4.

**Three Factor Eating Questionnaire**

The Three Factor Eating Questionnaire contains 18 questions, each of which is scored from 1 to 4, with higher values indicating a higher level of the behaviour. Domain scores (Cognitive Restraint, Uncontrolled Eating, and Emotional Eating) are calculated as the mean of all the questions within a domain.

The table below indicates which questions are included in which domain:

| **Domain** | **Questions included in domain** |
| --- | --- |
| Cognitive Restraint | 2, 11, 12, 15, 16, 18 |
| Uncontrolled Eating | 1, 4, 5, 7, 8, 9, 13, 14, 17 |
| Emotional Eating | 3, 6, 10 |

The table below indicates how each question is scored:

| **Question** | **Scoring system** |
| --- | --- |
| Q1 to Q13 | Definitely true = 4  Mostly true = 3  Mostly false = 2  Definitely false = 1 |
| Q14 | Almost always = 4  Often between meals = 3  Sometimes between meals = 2  Only at meal times = 1 |
| Q15 | Almost always = 4  Usually = 3  Seldom = 2  Almost never = 1 |
| Q16 | Very likely = 4  Moderately likely = 3  Slightly likely = 2  Unlikely = 1 |
| Q17 | At least once a week likely = 4  Sometimes likely = 3  Rarely likely = 2  Never = 1 |
| Q18 | Answer 7-8 = 4  Answer 5-6 = 3  Answer 3-4 = 2  Answer 1-2 = 1 |
