# Supplementary material for: Randomised controlled trial and economic evaluation of a task-based weight management group programme
Source: BMC Public Health. 2019 Apr 2;19:365. doi: 10.1186/s12889-019-6679-3 (PMC6444848; doi:10.1186/s12889-019-6679-3)
Supplement: Supplementary file 3 — Statistical Analysis Plan (SAP). Statistical Analysis Plan Version 2.0 14th April 2015. (DOCX 38 kb) [file 12889_2019_6679_MOESM3_ESM.docx]

**Additional file 3 –Secondary outcomes**

## Secondary outcomes

##### Changes in BMI

As expected change in BMI followed change in weight, with participants in the WAP arm showing a greater reduction in BMI that those in the nurse arm (see Table 1).

##### Changes in waist circumference

Table 2 shows participants in the nurse arm showed a greater reduction in waist circumference than those in the WAP arm at the end of treatment (-7.7 vs. -3.9 cm, p=0.001), but this was reversed at 6-month follow-up (-1.5 vs. -5.0 cm, p=0.004). By 12 months the difference had narrowed (-2.0 vs. -4.1 cm) and the difference was no longer significant (p=0.07).

##### Proportion of participants losing at least 5% or 10% of their baseline body weight

At 12-month follow-up a significantly greater proportion of participants in the WAP arm had lost at least 5% of body weight, compared with participants in the nurse arm (41% vs. 27%, p=0.004). Similarly a higher proportion of participants in WAP lost 10% of their baseline body weight than those in the nurse arm (participants in WAP were twice as likely as those in the nurse arm to have lost 10% of their weight), but the difference was not significant (see **Table 3**).

##### Changes in blood pressure

The only significant change in blood pressure between the arms was a greater drop in systolic blood pressure in the nurse arm at the end of treatment (-9.6 vs. -2.1 mmHg, p=0.02). At 12 month follow up there was no significant difference in blood pressure between participants in the nurse and WAP arms (see Table 4).

Table 1 – Change in BMI

|  | Nurse arm  (N=109)  Mean (SD)^1^ | WAP arm  (N=221)  Mean (SD)^1^ | Treatment effect (95% CI) ^2^ | | P-value |
| --- | --- | --- | --- | --- | --- |
| Change in BMI: mean (SD) | | | |  | |
| 1 month | -0.4 (0.6) | -0.4 (0.6) | 0.0 (-0.2, 0.2) | | 0.73 |
| 2 months | -0.8 (0.9) | -1.2 (1.0) | -0.4 (-0.6, -0.1) | | 0.005 |
| 6 months | -0.7 (1.5) | -1.8 (1.9) | -0.9 (-1.4, -0.5) | | <0.001 |
| 12 months | -0.8 (2.3) | -1.5 (2.6) | -0.7 (-1.3, 0.0) | | 0.04 |

^1^ The summary statistics (mean and SD) were calculated based on the number of participants with a recorded outcome at 1, 2, 6 and 12 months, which were as follows: for the nurse arm, 74, 62, 70, and 83, respectively; for the WAP arm, 164, 144, 141, and 149, respectively.

^2^ Treatment effects are presented as a difference in means (estimated from a mixed-effects regression model) between the two arms. Calculation is based on data from 97 participants in the nurse arm and 194 participants in the WAP arm.

Table 2 – Change in waist circumference

|  | Nurse arm  (N=109)  Mean (SD)^1^ | WAP arm  (N=221)  Mean (SD)^1^ | Treatment effect (95% CI) ^2^ | | P-value |
| --- | --- | --- | --- | --- | --- |
| Change in waist circumference (cm): mean (SD) | | | |  | |
| 2 months | -7.7 (7.3) | -3.9 (4.9) | 3.9 (2.0, 5.7) | | 0.001 |
| 6 months | -1.5 (6.2) | -5.0 (6.7) | -3.1 (-5.1, -1.2) | | 0.004 |
| 12 months | -2.0 (7.3) | -4.1 (7.9) | -2.0 (-4.1, 0.2) | | 0.07 |

^1^ The summary statistics (mean and SD) were calculated based on the number of participants with a recorded outcome at 2, 6 and 12 months, which were as follows: for the nurse arm, 60, 70, and 83, respectively; for the WAP arm, 140, 141, and 149, respectively.

^2^ Treatment effects are presented as a difference in means (estimated from a mixed-effects regression model) between the two arms.

**Table 3 – Participants losing 5% and 10% of baseline body weight**

|  | Nurse arm  (N=109)  N (%)^1^ | WAP arm  (N=221)  N (%)^1^ | Odds Ratio (95% CI) ^2^ | | | P-value |
| --- | --- | --- | --- | --- | --- | --- |
| Participants losing 5% of their body weight | | | |  | | |
| 2 months | 10 (16) | 32 (23) | 2.41 (0.69, 8.46) | | | 0.17 |
| 6 months | 14 (20) | 65 (46) | 31.60 (6.52, 153.18) | | | <0.001 |
| 12 months | 22 (27) | 61 (41) | 14.61 (2.32, 91.96) | | | 0.004 |
| Participants losing 10% of their body weight | | | |  | | |
| 2 months | 0 (0) | 2 (1) | - ^3^ | | - | |
| 6 months | 3 (4) | 26 (18) | 5.10 (1.48, 17.56) | | 0.01 | |
| 12 months | 7 (8) | 25 (17) | 2.50 (0.99, 6.32) | | 0.05 | |

^1^ The summary statistics (N and %) were calculated based on the number of participants with a recorded outcome at 1, 2, 6 and 12 months, which were as follows: for the nurse arm, 74, 62, 70, and 83, respectively; for the WAP arm, 164, 144, 141, and 149, respectively.

^2^ Calculation is based on data from 97 participants in the nurse arm and 194 participants in the WAP arm.

^3^ There were only two events at two months; we therefore did not perform an analysis at this time point.

Table 4 – Change in blood pressure

|  | Nurse arm  (N=109)  Mean (SD)^1^ | WAP arm  (N=221)  Mean (SD)^1^ | Treatment effect  (95% CI) ^2^ | | P-value |
| --- | --- | --- | --- | --- | --- |
| Change in systolic blood pressure – mean (SD) | | | |  | |
| 2 months | -9.6 (14.4) | -2.1 (13.7) | 5.6 (1.0, 10.3) | | 0.02 |
| 6 months | -5.1 (13.0) | -5.1 (14.6) | 0.4 (-4.4, 5.1) | | 0.88 |
| 12 months | -3.5 (16.0) | -2.8 (15.0) | 0.6 (-4.3, 5.4) | | 0.81 |
| Change in diastolic blood pressure – mean (SD) | | | |  | |
| 2 months | -0.6 (8.9) | -1.5 (7.7) | -0.3 (-3.0, 2.4) | | 0.81 |
| 6 months | -2.0 (9.2) | -3.6 (8.4) | -0.9 (-3.8, 1.9) | | 0.51 |
| 12 months | -0.4 (10.2) | -1.7 (9.1) | -0.7 (-3.6, 2.1) | | 0.59 |

^1^ The summary statistics (mean and SD) were calculated based on the number of participants with a recorded outcome at 2, 6 and 12 months, which were as follows: for the nurse arm, 60, 70, and 83, respectively; for the WAP arm, 140, 141, and 149, respectively.

^2^ Treatment effects are presented as a difference in means (estimated from a mixed-effects regression model) between the two arms.

##### Changes in food knowledge

Participants in the WAP arm showed a significant increase in their knowledge of calorie content of foods, compared to participants in the nurse arm, at the end of treatment and at 6-month follow-up (see Table 5). By 12-month follow-up this effect had disappeared.

##### Changes in food craving

All participants showed a decrease in the frequency and strength of food craving, at 1, 2, 6 and 12-month follow-up points (see Table 5). There were no significant differences between the groups.

##### Changes in three-factor eating questionnaires

Changes in the domains of the Three Factor Eating Questionnaire were minimal (see Table 5). Participants in both arms showed a small increase in cognitive restraint scores, but the increase was greater in the WAP arm compared with the nurse arm at the end of treatment (0.4 versus 0.2, p=0.05). Participants, on average, showed small decreases in uncontrolled and emotional eating scores. There were no significant differences in the changes between study arms.

##### Changes in levels of physical activity

Participants in both arms increased their levels of physical activity above baseline across the duration of the study to the same extent (see Table 6) (818 vs. 264 MET-minutes/week, p=0.09).

Participants reported reducing their sitting time by an hour at the end of treatment, but no significant differences between groups was observed.

Table 5 – Changes in food knowledge, craving and eating

|  | Nurse arm  Mean (SD)^1^ | WAP arm  Mean (SD)^1^ | Treatment effect  (95% CI) ^2^ | P-value |
| --- | --- | --- | --- | --- |
| Change in Food Knowledge Assessment Questionnaire score | | | | |
| 2 months | 0.1 (1.7) | 1.1 (1.7) | 1.1 (0.6, 1.6) | <0.001 |
| 6 months | 0.2 (2.0) | 0.8 (1.7) | 0.5 (0.1, 1.0) | 0.03 |
| 12 months | 0.4 (1.9) | 0.6 (2.0) | 0.1 (-0.3, 0.6) | 0.61 |
| Change in Food Craving Inventory score (Frequency domain) | | | | |
| 1 month | -2.2 (3.8) | -2.1 (3.6) | -0.3 (-1.4, 0.7) | 0.53 |
| 2 months | -1.8 (3.8) | -2.0 (3.8) | -0.6 (-1.7, 0.5) | 0.25 |
| 6 months | -1.2 (4.1) | -1.7 (3.7) | -0.8 (-1.9, 0.3) | 0.13 |
| 12 months | -0.9 (3.7) | -1.5 (4.0) | -0.8 (-1.9, 0.2) | 0.12 |
| Change in Food Craving Inventory score (Strength domain) | | | | |
| 1 month | -2.3 (4.4) | -2.0 (3.4) | 0.1 (-0.9, 1.1) | 0.85 |
| 2 months | -2.2 (4.0) | -1.7 (3.9) | -0.1 (-1.1, 0.9) | 0.85 |
| 6 months | -1.2 (3.7) | -1.3 (4.0) | -0.3 (-1.4, 0.7) | 0.48 |
| 12 months | -1.4 (3.8) | -1.3 (4.2) | -0.2 (-1.2, 0.9) | 0.75 |
| Change in Three Factor Eating Questionnaire score (Cognitive Restraint domain) | | | | |
| 2 months | 0.2 (0.6) | 0.4 (0.6) | 0.2 (0.0, 0.3) | 0.05 |
| 6 months | 0.2 (0.5) | 0.4 (0.7) | 0.1 (0.0, 0.3) | 0.07 |
| 12 months | 0.2 (0.6) | 0.3 (0.6) | 0.1 (0.0, 0.3) | 0.10 |
| Change in Three Factor Eating Questionnaire score (Uncontrolled Eating domain) | | | | |
| 2 months | -0.2 (0.5) | -0.1 (0.5) | 0.1 (-0.1, 0.2) | 0.35 |
| 6 months | -0.2 (0.5) | -0.2 (0.5) | 0.0 (-0.1, 0.1) | 0.93 |
| 12 months | -0.3 (0.6) | -0.2 (0.6) | 0.0 (-0.1, 0.2) | 0.66 |
| Change in Three Factor Eating Questionnaire score (Emotional Eating domain) | | | | |
| 2 months | -0.3 (0.8) | -0.2 (0.7) | 0.1 (-0.1, 0.3) | 0.32 |
| 6 months | -0.3 (0.7) | -0.2 (0.7) | 0.1 (-0.2, 0.3) | 0.59 |
| 12 months | -0.3 (0.7) | -0.2 (0.7) | 0.1 (-0.1, 0.2) | 0.54 |

^1^ The summary statistics (mean and SD) were calculated based on the number of participants with a recorded outcome at each time point.

^2^ Treatment effects are presented as a difference in means (estimated from a mixed-effects regression model) between the two arms.

Table 6 – Changes in International Physical Activity Questionnaire scores

|  | Nurse arm  Median (IQR)^1^ | WAP arm  Median (IQR)^1^ | Treatment effect  (95% CI) ^2^ | P-value |
| --- | --- | --- | --- | --- |
|  | | | | |
| MET-minutes/week | | |  |  |
| 2 months | 264 (-347, 1030) | 818 (0, 2517) | 923 (-167, 2014) | 0.09 |
| 6 months | 336 (-240, 1644) | 415(-258, 1584) | -441 (-1380, 497) | 0.33 |
| 12 months | 215 (-763, 1589) | 359(-385, 1750) | 613 (-312, 1537) | 0.18 |
| Minutes spent sitting/day | | |  |  |
| 2 months | -60 (-120, 0) | -60 (-150, 60) | -12 (-93, 69) | 0.77 |
| 6 months | 0 (-90, 30) | -60 (-150, 0) | -5 (-71, 61) | 0.87 |
| 12 months | -60 (-120, 60) | 0 (-120, 60) | 19 (-51, 89) | 0.57 |

^1^ The summary statistics (median and IQR) were calculated based on the number of participants with a recorded outcome at each time point.

^2^ Treatment effects are presented as a difference in means (estimated from a mixed-effects regression model) between the two arms.

## Adverse events

Table 7 provides a summary of all adverse events. There were more AEs in the WAP group, although this difference was not statistically significant (WAP 11% vs. nurse 6%; OR=2.19, 95%CI: 0.86 – 5.58, p=0.10).

Three serious adverse events were reported, where participants were hospitalised overnight for shortness of breath, myalgia, and gastrointestinal complaints. These were all in the WAP arm, and were not related to study procedures.

Table 7 – Summary of adverse events

|  | Nurse arm (n=109) | WAP arm (n=221) |
| --- | --- | --- |
| Number of adverse events* | 8 | 45 |
| Number of patients with at least one adverse event | 6 | 25 |
| Number of adverse events per patient |  |  |
| 0 | 103 | 196 |
| 1 | 5 | 16 |
| 2 | 0 | 5 |
| 3 | 1 | 1 |
| 4 | 0 | 0 |
| 5 | 0 | 2 |
| 6 | 0 | 1 |
| Number of serious adverse events | 0 | 3 |
| Systems affect by adverse event |  |  |
| Gastrointestinal | 5 | 21 |
| Nervous system | 0 | 7 |
| General disorders | 0 | 6 |
| Musculoskeletal and connective tissue | 0 | 6 |
| Psychiatric | 0 | 2 |
| Respiratory, thoracic and mediastinal | 1 | 1 |
| Infections and infestations | 2 | 0 |
| Blood and lymphatic | 0 | 1 |
| Skin and subcutaneous tissue | 0 | 1 |
| Adverse event category |  |  |
| Arthralgia | 0 | 4 |
| Bloating | 0 | 2 |
| Bruising | 0 | 1 |
| Constipation | 1 | 2 |
| Diarrhoea | 1 | 6 |
| Dizziness | 0 | 4 |
| Dry skin | 0 | 1 |
| Flatulence | 0 | 2 |
| Flu like symptoms | 0 | 6 |
| Generalized muscle weakness | 0 | 1 |
| Headache | 0 | 2 |
| Haemorrhoids | 1 | 0 |
| Insomnia | 0 | 2 |
| Lung infection | 2 | 0 |
| Memory impairment | 0 | 1 |
| Myalgia | 0 | 1 |
| Steatorrhoea | 0 | 7 |
| Stomach pain | 1 | 1 |
| Voice alteration | 0 | 1 |
| Vomiting | 1 | 1 |
| Wheezing | 1 | 0 |

*Some patients experienced more than one adverse event
